# MOF-associated complexes ensure stem cell identity and *Xist* repression

**DOI:** 10.7554/eLife.02024

**Published:** 2014-05-19

**Authors:** Tomasz Chelmicki, Friederike Dündar, Matthew James Turley, Tasneem Khanam, Tugce Aktas, Fidel Ramírez, Anne-Valerie Gendrel, Patrick Rudolf Wright, Pavankumar Videm, Rolf Backofen, Edith Heard, Thomas Manke, Asifa Akhtar

**Affiliations:** 1 Department of Chromatin Regulation, Max Planck Institute of Immunobiology and Epigenetics, Freiburg, Germany; 2 Faculty of Biology, University of Freiburg, Freiburg, Germany; 3 Bioinformatics Department, Max Planck Institute for Immunobiology and Epigenetics, Freiburg, Germany; 4 Mammalian Developmental Epigenetics Group, Institute Curie, Paris, France; 5 Bioinformatics Group, Department of Computer Science, University of Freiburg, Freiburg, Germany; 6 BIOSS Center for Biological Signalling Studies, University of Freiburg, Freiburg, Germany; 7 Center for Biological Systems Analysis, University of Freiburg, Freiburg, Germany; 8 Center for Non-Coding RNA in Technology and Health, University of Copenhagen, Frederiksberg, Denmark; Howard Hughes Medical Institute, New York University School of Medicine, United States

**Keywords:** *D. melanogaster*, epigenetics, chromatin, transcription, acetylation, X inactivation, mouse

## Abstract

Histone acetyl transferases (HATs) play distinct roles in many cellular processes and are frequently misregulated in cancers. Here, we study the regulatory potential of MYST1-(MOF)-containing MSL and NSL complexes in mouse embryonic stem cells (ESCs) and neuronal progenitors. We find that both complexes influence transcription by targeting promoters and TSS-distal enhancers. In contrast to flies, the MSL complex is not exclusively enriched on the X chromosome, yet it is crucial for mammalian X chromosome regulation as it specifically regulates *Tsix*, the major repressor of Xist lncRNA. MSL depletion leads to decreased *Tsix* expression, reduced REX1 recruitment, and consequently, enhanced accumulation of *Xist* and variable numbers of inactivated X chromosomes during early differentiation. The NSL complex provides additional, *Tsix*-independent repression of *Xist* by maintaining pluripotency. MSL and NSL complexes therefore act synergistically by using distinct pathways to ensure a fail-safe mechanism for the repression of X inactivation in ESCs.

**DOI:** http://dx.doi.org/10.7554/eLife.02024.001

## eLife digest

Gene expression is controlled by a complicated network of mechanisms involving a wide range of enzymes and protein complexes. Many of these mechanisms are identical in males and females, but some are not. Female mammals, for example, carry two X chromosomes, whereas males have one X and one Y chromosome. Since the two X chromosomes in females contain essentially the same set of genes, one of them undergoes silencing to prevent the overproduction of certain proteins. This process, which is called X-inactivation, occurs during different stages of development and it must be tightly controlled.

An enzyme called MOF was originally found in flies in two distinct complexes—the male-specific lethal (MSL) complex, which forms only in males, and the non-specific lethal (NSL) complex, which is ubiquitous in both males and females. These complexes are evolutionary conserved and are also found in mammals. While mammalian MOF is reasonably well understood, the MSL and NSL complexes are not, so Chelmicki, Dündar et al. have used various sequencing techniques, in combination with biochemical experiments, to investigate their roles in embryonic stem cells and neuronal progenitor cells in mice.

These experiments show that MSL and NSL complexes engage in the regulation of thousands of genes. Although the two complexes often show different gene preferences, they often regulate the same cellular processes. The MSL/NSL-dependent regulation of X chromosome inactivation is a prime example of this phenomenon.

The MSL complex reduces the production of an RNA molecule called Xist, which is responsible for the inactivation of one of the two X chromosomes in females. The NSL complex, meanwhile, ensures the production of multiple proteins that are crucial for the development of embryonic stem cells, and are also involved in the repression of X inactivation.

This analysis sheds light on how different complexes can cooperate and complement each other in order to reach the same goal in the cell. The knowledge gained from this study will pave the way towards better understanding of complex processes such as embryonic development, organogenesis and the pathogenesis of disorders like cancer.

**DOI:** http://dx.doi.org/10.7554/eLife.02024.002

## Introduction

Histone acetyl transferases (HATs) are among the key architects of the cellular epigenetic landscape as the acetylation of histones is unanimously associated with transcriptionally active domains. Many HATs also have the ability to acetylate non-histone proteins extending their influence to diverse cellular pathways inside and outside of the nucleus (reviewed in Sapountzi and Cote, 2011). Based on their catalytic domains, the HATs are classified into two major families, GCN5 N-acetyl transferases (GNATs) and MYST HATs (named after the founding members MOZ, Ybf2/Sas3, Sas2, Tip60), that encompass diverse sets of protein complexes. The individual complex members enhance and modulate the enzymes' activities, guiding the versatile HATs towards specific functions. GCN5, for example, is part of SAGA, ATAC, and SLIK complexes that are associated with distinct histone tail modifications and differential gene regulation (reviewed in Lee and Workman, 2007; Nagy et al., 2010). In contrast, one of the well-known members of the MYST family, MOF (also known as: KAT8, MYST1), is rather substrate-specific for lysine 16 of histone H4 (H4K16) (Akhtar and Becker, 2000) and its interaction partners are thought to mainly alter the specificity and extent of MOF's H4K16 acetylation (H4K16ac). As part of the male-specific lethal (MSL) complex (MSL1, MSL2, MSL3, MOF, MLE, roX1 and roX2 lncRNAs) in *Drosophila melanogaster*, MOF is recruited to the single X chromosome of male flies. The subsequent spreading of H4K16 acetylation results in transcriptional upregulation of the male X chromosome, the major means of *D. melanogaster* dosage compensation (reviewed in Conrad and Akhtar, 2011). In addition to the highly specialized MSL-associated role, MOF is also involved in the more universal and sex-independent regulation of housekeeping genes within the non-specific lethal (NSL) complex (NSL1, NSL2, NSL3, MBD-R2, MCRS2, MOF, WDS) (Mendjan et al., 2006; Raja et al., 2010; Feller et al., 2012; Lam et al., 2012).

MOF and most of its interaction partners are conserved in mammals, where MOF is also responsible for the majority of H4K16 acetylation (Smith et al., 2005; Taipale et al., 2005). MOF is essential for mammalian embryonic development and unlike the male-specific lethality in *Drosophila*, deletion of *Mof* in mice is lethal for both sexes (Gupta et al., 2008; Thomas et al., 2008). More specifically, mammalian MOF is critical for physiological nuclear architecture (Thomas et al., 2008), DNA damage repair (Gupta et al., 2008), maintenance of stem cell pluripotency (Li et al., 2012), differentiation of T cells (Gupta et al., 2013), and survival of post-mitotic Purkinje cells (Kumar et al., 2011). Compared to MOF, mammalian MSL and NSL complex members are poorly understood. Nevertheless, the individual complex members appear to have important functions in vivo as mutations of the NSL complex member KANSL1 cause the core phenotype of the 17q21.31 microdeletion syndrome (Koolen et al., 2012; Zollino et al., 2012) and are common amongst patients with both Down syndrome and myeloid leukemia (Yoshida et al., 2013). Another NSL-associated protein, PHF20 has been shown to associate with methylated Lys370 and Lys382 of p53 (Cui et al., 2012) and to be required for somatic cell reprogramming (Zhao et al., 2013a). WDR5 was shown to be an essential regulator of the core transcription network in embryonic stem cells (Ang et al., 2011). The mammalian counterpart of *Drosophila* MSL2 was shown to have the capacity to ubiquitylate p53 (Kruse and Gu, 2009) and lysine 34 of histone 2B (Wu et al., 2011).

In the study presented here, we set out to dissect the mammalian MOF functions within the MSL and NSL complexes using genome-wide chromatin immunoprecipitation and transcriptome profiles and biochemical experiments for the core members of MSL and NSL complexes in mouse embryonic stem cells (ESCs) and neuronal progenitor cells (NPCs). We found that the MSL and NSL members possess concurrent, as well as independent functions and that effects generally attributed to MOF are frequently accompanied by the NSL complex. The NSL complex abundantly binds to promoters of broadly expressed genes in ESCs and NPCs. These genes are predominantly downregulated upon depletion of either MOF or KANSL3. In contrast, the MSL complex shows more restricted binding in ESCs, which expands after differentiation, particularly at NPC-specific genes. In addition to promoter-proximal binding, we discover several thousand binding sites of KANSL3 and MSL2 at promoter-distal loci with enhancer-specific epigenetic signatures. The majority of these distal regulatory sites are bound in ESCs, but not in differentiated cells, and genes that are predicted to be targeted by TSS-distal binding of MSL2 are frequently downregulated in sh*Msl2*-treated cells. The distinct, yet synergistic actions of both complexes become very apparent at the X inactivation center (XIC) that encodes numerous non-coding RNAs involved in the silencing of one of the two X chromosomes in differentiating female cells. We show that the MSL but not the NSL complex directly promotes expression of *Tsix*, the inverse transcript and the key murine repressor of *Xist* during early differentiation. Depletion of MSL proteins results in attenuation of *Tsix* transcription, enhanced *Xist* RNA accumulation and ‘chaotic’ inactivation of variable numbers of X chromosomes during early differentiation. In addition to the very specific effect of MSL1/MSL2-depletion on the XIC genes, we show that MOF together with the NSL complex also influences *Xist* levels, but instead of affecting *Tsix*, MOF and KANSL3 depletion diminish key pluripotency factors involved in repressing *Xist*. Our study provides novel insights into the intricate interplay between MSL and NSL complexes in orchestrating gene expression. Furthermore, we demonstrate how MSLs and NSLs ensure the active state of two X chromosomes in mouse embryonic stem cells via distinct mechanisms.

## Results

### MOF and its complexes show distinct chromatin binding dynamics during differentiation

**Figure 1.**
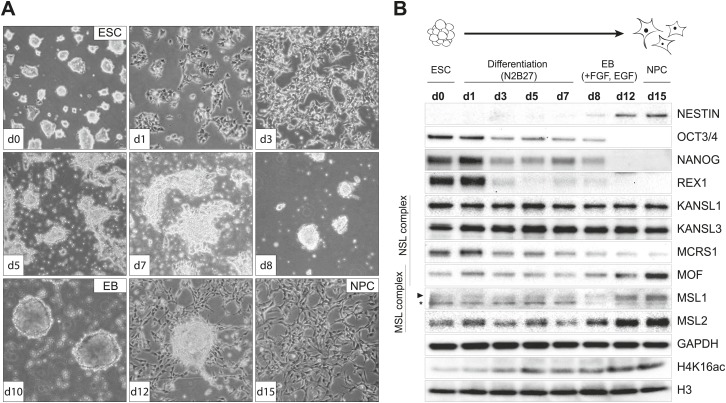
Distinct dynamics of MOF, MSL and NSL complexes during differentiation from ESCs to NPCs. (**A**) We monitored the cell morphology during differentiation of mouse embryonic stem cells into neuronal progenitor cells (NPC) via embryoid body formation (EB) with bright field microscopy. The day of differentiation is indicated in white boxes. (**B**) Western blot analysis for ESC to NPC differentiation. Stages of differentiation together with the day of differentiation (d0–d15) are indicated on top. GAPDH and histone 3 (H3) were used as loading controls. For expression analysis see Figure 1—figure supplement 1. **DOI:** http://dx.doi.org/10.7554/eLife.02024.003

To examine the behavior of MSL and NSL proteins in a cell type-specific manner, we derived homogeneous populations of multipotent neuronal progenitor cells (NPCs) from mouse embryonic stem cells (ESCs) (Conti et al., 2005; Splinter et al., 2011; Gendrel et al., 2014). We followed the progress of the differentiation process by monitoring cell morphology (Figure 1A), as well as protein (Figure 1B) and transcript levels of ESC- and NPC-specific markers (Figure 1—figure supplement 1A–C). To gain a better understanding of how MOF-associated complexes behave throughout the differentiation process, in parallel to cell type-specific markers, we also monitored the RNA and protein levels of MOF, MSL (MSL1, MSL2), and NSL (KANSL1, KANSL3, MCRS1) complex members (Figure 1B, Figure 1—figure supplement 1A). Interestingly, MSL and NSL complex members showed distinct RNA and protein dynamics during the process of differentiation: KANSL1 and KANSL3 protein levels remained unchanged, whereas MSL1, MSL2 and MOF became more abundant in NPCs accompanied by increased H4K16 acetylation (H4K16ac) (Figure 1B). These results were confirmed using another ES cell line and its NPC derivative (Figure 1—figure supplement 1D). The specificities of the antibodies were confirmed by co-immunoprecipitation assays (Figure 1—figure supplement 2A–C), as well as shRNA-mediated knockdowns followed by western blot analyses (for individual knockdowns please see below).

**Figure 1—figure supplement 1.**
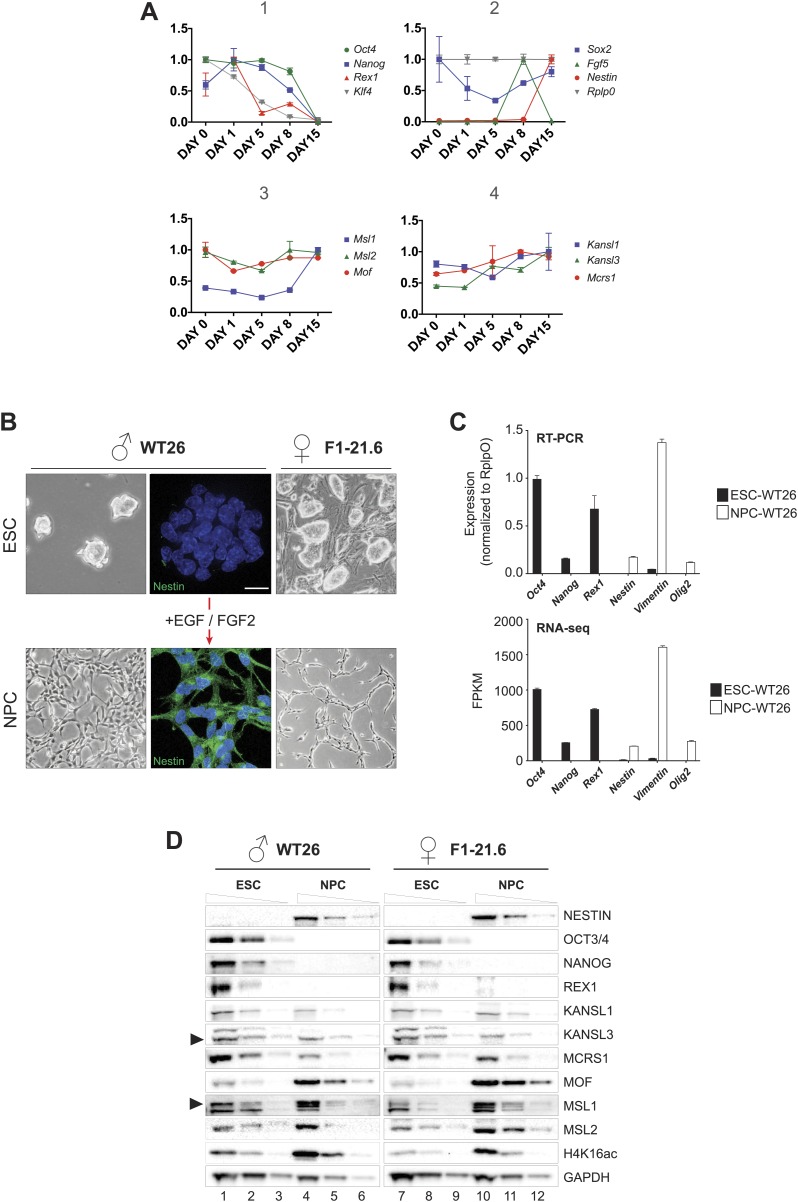
Monitoring RNA and protein levels in ESCs and NPCs. (**A**) We monitored the expression dynamics during ESC differentiation for markers of pluripotency (*Oct4, Nanog, Rex1, Klf4*), embryoid body formation (*Fgf*5), differentiation (*Sox2*), and NPC (*Nestin*). Panels 3 and 4 contain the expression profiles for members of the MSL complex (*Msl1*, *Msl2*), *Mof*, and the NSL complex (*Kansl1*, *Kansl3*, *Mcrs1*), respectively. All results are represented as relative values individually normalized to *Rplp0* expression levels (panel 2) on a given day and to the highest expression level of a given gene during the entire differentiation process (highest expression level of each gene = 1). The x-axes show days of differentiation. All results are expressed as means ± SD for technical replicates. For primers see Supplementary file 3C. (**B**) Bright field images illustrate the cell morphology before and after the process of differentiation. The immunofluorescence analysis indicates the specific staining for the NESTIN (green) in neuronal progenitors (NPC); DNA is counterstained with DAPI (blue). (**C**) Expression changes for selected ESC-specific and NPC-specific markers before and after differentiation of wild-type WT26 cells using RT-PCR analysis and RNA-seq. (**D**) Western blots for proteins from two ES cell lines and their NPC derivatives. Different dilutions were loaded (100%, 30%, 10%) with the order indicated on top of the blots. Anti-GAPDH was used as loading control; arrows indicate the protein of interest. **DOI:** http://dx.doi.org/10.7554/eLife.02024.004

**Figure 1—figure supplement 2.**
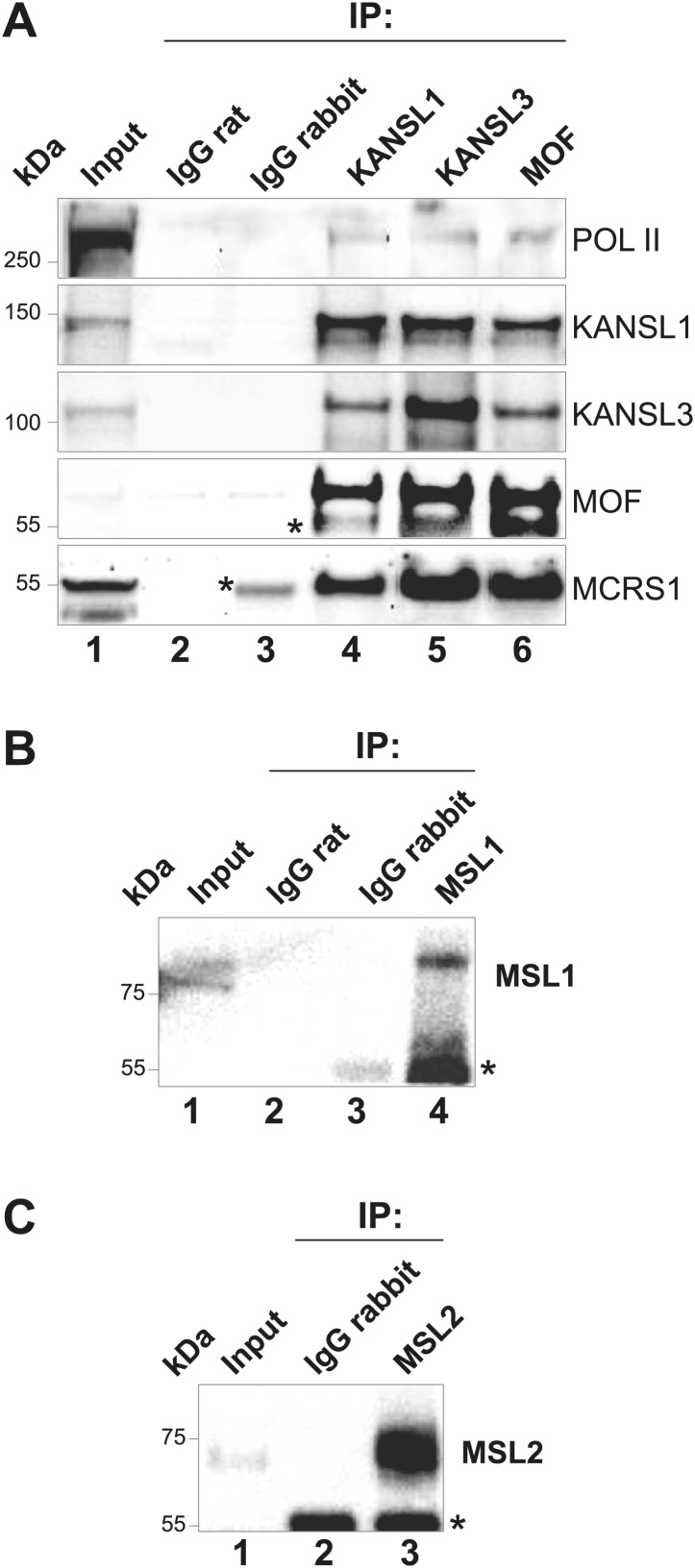
Verification of antibodies used in this study. (**A**) Immunoprecipitations from ESC nuclear extracts with antibodies specific for KANSL1, KANSL3 or MOF, and rabbit or rat antisera. The blot was probed with indicated antibodies showing the co-immunoprecipitation of several NSL complex members. Pol II = RNA Polymerase II. (**B**) and (**C**) same as (**A**) except that immunoprecipitations were performed with antibodies specific to MSL1 (**B**) and MSL2 (**C**). Asterisks represent the IgG signal. **DOI:** http://dx.doi.org/10.7554/eLife.02024.005

**Figure 2.**
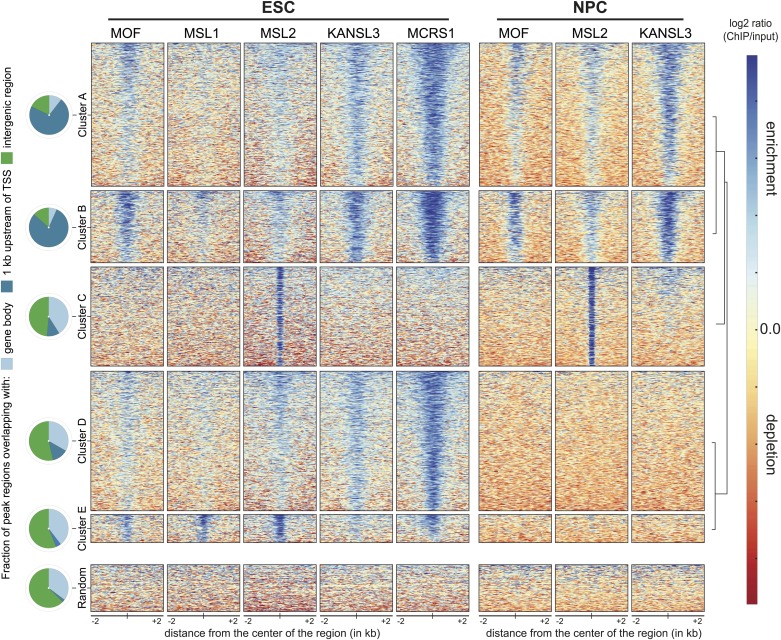
Distinct and shared binding sites of MOF and its complexes in mouse ESCs and NPCs. We applied unsupervised clustering on the union of peaks from all ChIP-seq samples and thereby identified five distinct groups of binding for MOF, MSL and NSL proteins in ESCs and NPCs. Shown here are the input-normalized ChIP signals for each cluster of peaks including a size-matched control set of random genomic regions. The order of the regions is the same for all columns. The pie charts on the left indicate the number of regions from each cluster that overlap with gene bodies, the region 1 kb upstream of genes' TSS or intergenic regions. **DOI:** http://dx.doi.org/10.7554/eLife.02024.006

To assess the distinct behaviors of the complexes in more detail, we generated genome-wide chromatin binding profiles for MSL1, MSL2 (MSL complex), KANSL3, MCRS1 (NSL complex), and MOF (MSL and NSL). ChIP-seq experiments in ESCs and NPCs (Figure 2) yielded large numbers of high-quality DNA sequence reads and excellent agreements between the biological replicates (Figure 2—figure supplement 1A, Supplementary file 1A). Using MACS for peak calling (Zhang et al., 2008) and additional stringent filtering (‘Materials and methods’), we scored between 1500 and 15,000 regions of significant enrichments for the different proteins (Supplementary file 1B).

**Figure 2—figure supplement 1.**
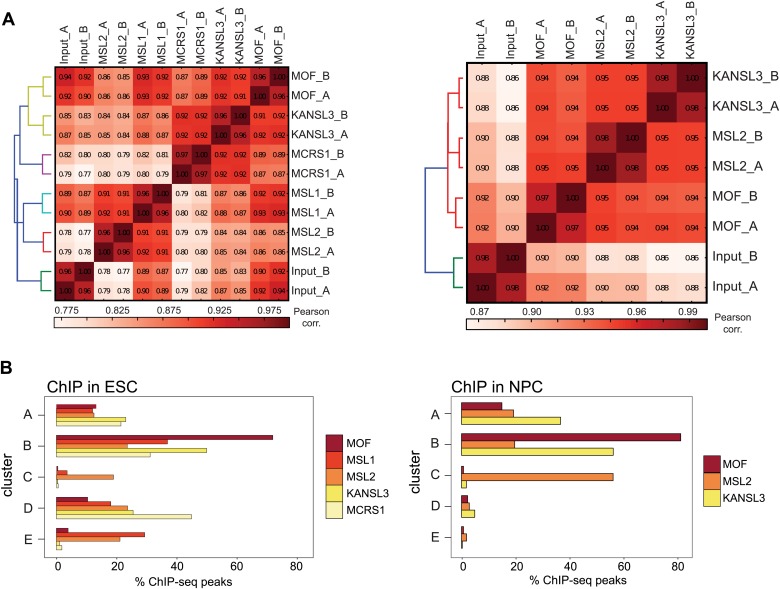
ChIP-seq quality measures. (**A**) Correlation plot for all individual ChIP-seq and input samples from ESCs (left) and NPCs. The genome was sampled in windows of 10 kb length; the numbers of reads per bin were counted for each ChIP sample and correlated using Pearson correlation. The calculation and heatmap visualization were done with the bamCorrelate module from the deepTools suite (Ramirez et al., 2014). (**B**) The bar chart depicts the fraction of ChIP-seq peaks for each protein that reside within each cluster shown in Figure 2, that is approximately 30% of MSL1 peaks in ESCs locate in cluster E. Note that the absolute numbers of peaks differ between the samples (see Supplementary file 1B for absolute peak numbers and ‘Materials and methods’ for peak calling details). **DOI:** http://dx.doi.org/10.7554/eLife.02024.007

To uncover patterns of co-occurrence and independent binding, we used unsupervised clustering on the input-normalized signals. This unbiased approach allowed us to determine five main groups of binding distinguished by different combinations of the proteins and cell-type-specific dynamics. As shown in Figure 2, three large clusters of binding sites encompassed regions, where at least 1 of the investigated proteins was present both in ESCs and NPCs (clusters A, B and C). The binding sites of clusters A and B predominantly overlapped with annotated transcription start sites (TSS) in contrast to the regions that were bound exclusively in ESCs, which tended to contain inter- and intragenic regions (clusters D and E, Figure 2). The width of the enrichments did not differ profoundly between the groups (cluster E: 836 bp median width, cluster A: 1782 bp median width). We found surprisingly few regions where MOF associated primarily with MSL complex members. Instead, approximately 80% of all MOF peaks displayed strong KANSL3 and MCRS1 signals (cluster B, see Figure 2 and Figure 2—figure supplement 1B), suggesting a predominant role of the NSL complex among MOF-associated complexes and a more specific role for the MSL complex at subsets of promoters and numerous intergenic and intronic regions. As the different clusters showed distinct enrichment patterns and diverse genomic localization, we set out to analyze the individual groups of binding in more detail.

### The MSL and NSL complexes co-occur on active promoters of constitutively expressed genes in ESCs and NPCs

**Figure 3.**
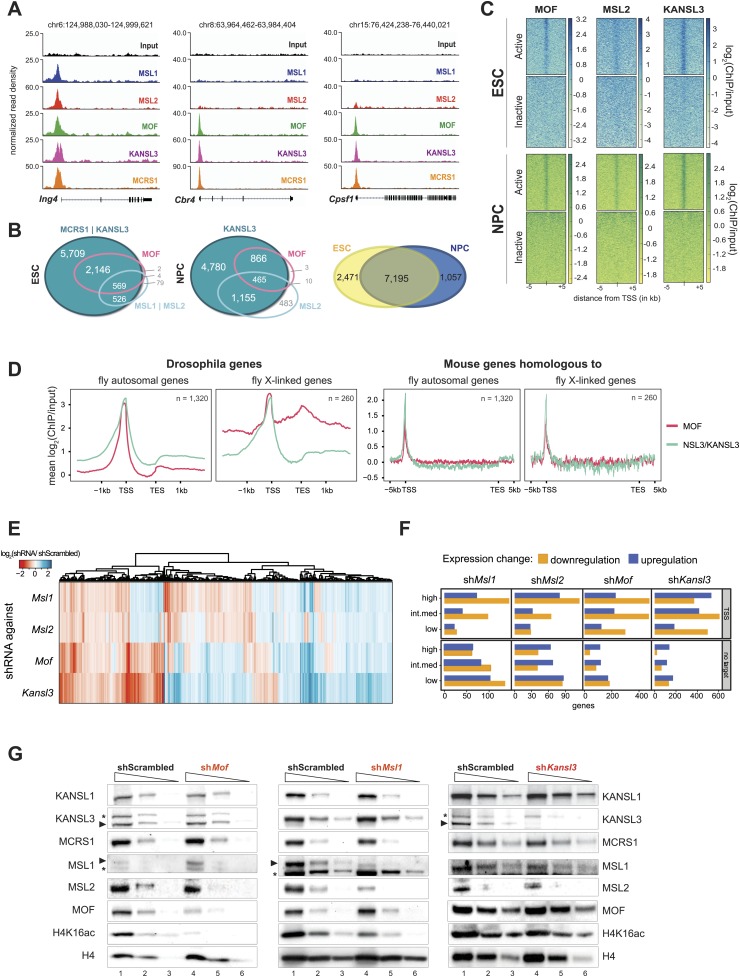
Both MOF-complexes bind to the TSS of broadly expressed genes in mouse ESCs and NPCs. (**A**) Genome browser snapshots of genes targeted by MSL and NSL complexes or by the NSL complex only. Signals were sequencing-depth-normalized and from ESCs. For ChIP-qPCR-based validation of the signals see Figure 3—figure supplement 4B. (**B**) Venn diagrams of genes whose promoter regions (TSS ± 500 bp) overlapped with ChIP-seq peaks of NSL complex members (KANSL3 and/or MCRS1), MOF and MSL complex members (MSL1 and/or MSL2). The right-most panel depicts the overlap of genes bound by at least one factor in ESCs and NPCs. (**C**) The heatmaps display the input-normalized ChIP enrichments of MOF, MSL2 and KANSL3 around the TSS of genes that were active in ESCs as well as NPCs based on RNA-seq data that we generated for both cell types. (**D**) Summary plots of genes bound by the NSL complex in *D. melanogaster* for which mouse homologues were found. The input-normalized ChIP-seq signals around the TSS reveal markedly increased binding of MOF for male X-linked fly genes (left panels) that was not recapitulated in the mouse (right panels; ChIP-seq signals from ESCs). Fly genes were scaled to 1.2 kb and values were extracted from published data sets, mouse genes were scaled to 30 kb. (**E**) Heatmap depicting results of RNA-seq experiments from different shRNA-treated cells. The colors correspond to log_2_ fold changes (shRNA-treated cells/scrambled control) for genes whose expression was significantly affected in all knockdown conditions. Values were ordered using hierarchical clustering. (**F**) Bar plot of gene counts for different gene classes. We determined significantly up- and downregulated genes for each knockdown condition and binned them according to their expression strength in wild-type ESCs (high, intermediate, low). Then, for each gene, information about the TSS-targeting was extracted from the corresponding ChIP-seq sample. Non-target genes are neither bound at the promoter nor the gene body and were not predicted to be regulated via TSS-distal binding sites in any of the 5 ChIP-seq ESC samples. For details on the target classification see ‘Materials and methods’. (**G**) Western blot analysis of MSL and NSL complex members and H4K16 acetylation in scrambled-, *Mof*-, *Msl1*-, and *Kansl3*-shRNA-treated male ESCs. Three concentrations (100%, 30%, 10%) of RIPA extract were loaded per sample. Asterisks mark the position of unspecific bands; triangles indicate the protein of interest. **DOI:** http://dx.doi.org/10.7554/eLife.02024.008

We first focused on the characterization of target promoters as the majority of MOF-binding was found around the TSS (mostly clusters A and B in Figure 2, Figure 3A). We identified 8947 TSSs overlapping with ChIP-seq peaks of KANSL3 and/or MCRS1 in ESCs that encompassed virtually all MOF- and MSL-bound TSSs (Figure 3B). This pattern did not change substantially in NPCs where TSSs overlapping with MOF peaks almost always (99%) showed significant enrichments of KANSL3 and in 35% of the cases additionally contained a peak of MSL2 (Figure 3B, middle panel). Genes that were TSS-bound in ESCs tended to be bound in NPCs as well (Figure 3B, middle panel and Figure 3—figure supplement 1A). We next generated RNA-seq data for ESCs and NPCs, determined genes that were expressed in both cell types (FPKM >4) and found that all ChIPed proteins preferably bound to the promoters of active genes (Figure 3C). Interestingly, in ESCs, genes whose TSSs were bound by members of both complexes showed higher median expression values than genes bound by only one complex (Figure 3—figure supplement 1B). In contrast to the differing expression values, analysis of gene ontology (GO) using DAVID (Huang da et al., 2009) revealed basic housekeeping functions for both gene groups, regardless of whether they were bound by the NSL complex only or by both MOF-complexes together (Figure 3—figure supplement 1C). Consistently, the promoters of all target gene groups were enriched for motifs associated with broad, non-cell-type-specific expression such as ELK1, YY1, CREB, and E2F (Xie et al., 2005; Farre et al., 2007) and showed profound enrichments of CpG islands (Figure 3—figure supplement 1D), which is indicative of housekeeping genes (Landolin et al., 2010). Interestingly, when we analyzed the subset of genes that gained binding of either KANSL3 or MSL2 in NPCs, we found strong enrichments of GO terms related to embryonic development for KANSL3 targets and cell migration and neuronal development for MSL2 targets.

**Figure 3—figure supplement 1.**
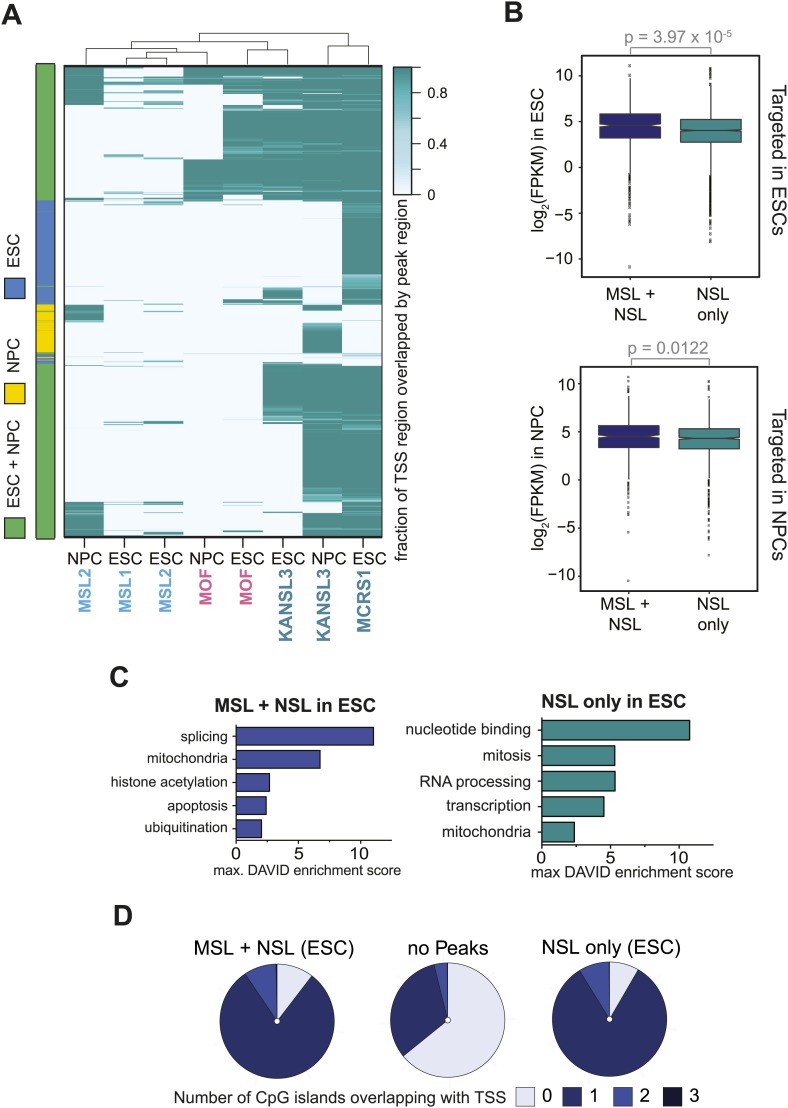
MSL and NSL complexes target promoters of broadly expressed genes in ESCs and NPCs. (**A**) The heatmap is related to Figure 3B as it is based on all genes that are bound by at least 1 ChIPed factor in ESCs or NPCs. The intensity of the color depicts the fraction of the 1 kb TSS-region that was covered by a binding site of MOF, MSL1, MSL2, KANSL3 or MCRS1. Rows and columns were sorted using hierarchical clustering on the Euclidean distances of the overlap fractions using R. The left color bar indicates which genes are targeted in 1 or both cell types. (**B**) Distribution of expression values from RNA-seq data in ESCs and NPCs for genes targeted by MSL and NSL complex members together or by the NSL complex only. p-values were calculated using Welch *t* test. (**C**) Results of the GO term analysis using DAVID (Huang da et al., 2009) on genes that were bound at the TSS in ESCs by NSL complex members only or both MSL and NSL complexes. (**D**) The pie charts depict how many times annotated TSSs overlapped with a CpG island. The vast majority of genes that were bound in ESCs by MSL and NSL together or by NSL complex members alone overlapped with at least 1 CpG island (dark and medium blue) while approximately 2/3 of the non-target-TSS did not overlap with any CpG island (light blue for 0 CpG islands within the queried regions). **DOI:** http://dx.doi.org/10.7554/eLife.02024.009

**Figure 3—figure supplement 2.**
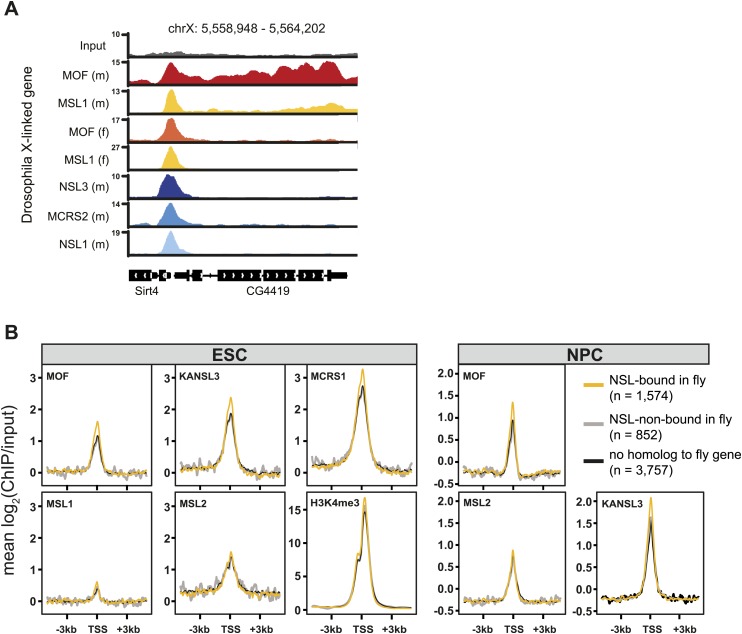
The NSL-, but not the MSL-binding mode of *D. melanogaster* is present in mammalian cells. (**A**) Exemplary genome browser snapshots of the X-linked fly gene CG4419. Shown here are the sequencing-depth normalized profiles for ChIP and corresponding input samples, clearly showing a broad enrichment of MOF and MSL1 along the entire gene body in male (m) *D. melanogaster* while all other marks show sharp enrichments around the TSS (including MSL1 and MOF in female (**F**) *D. melanogaster*) which are similar to those seen for both complexes in mouse cells (Figure 3A,D). (**B**) Comparison of expressed (FPKM >4) mouse genes whose homologous genes are either bound or not bound by MOF and its complexes in the fly. We extracted the input-normalized ChIP-seq values for 6 kb regions around the TSS using the computeMatrix module of deepTools (Ramirez et al., 2014). H3K4me3 signal is from a published data set, see Supplementary file 2 for the corresponding accession number. **DOI:** http://dx.doi.org/10.7554/eLife.02024.010

**Figure 3—figure supplement 3.**
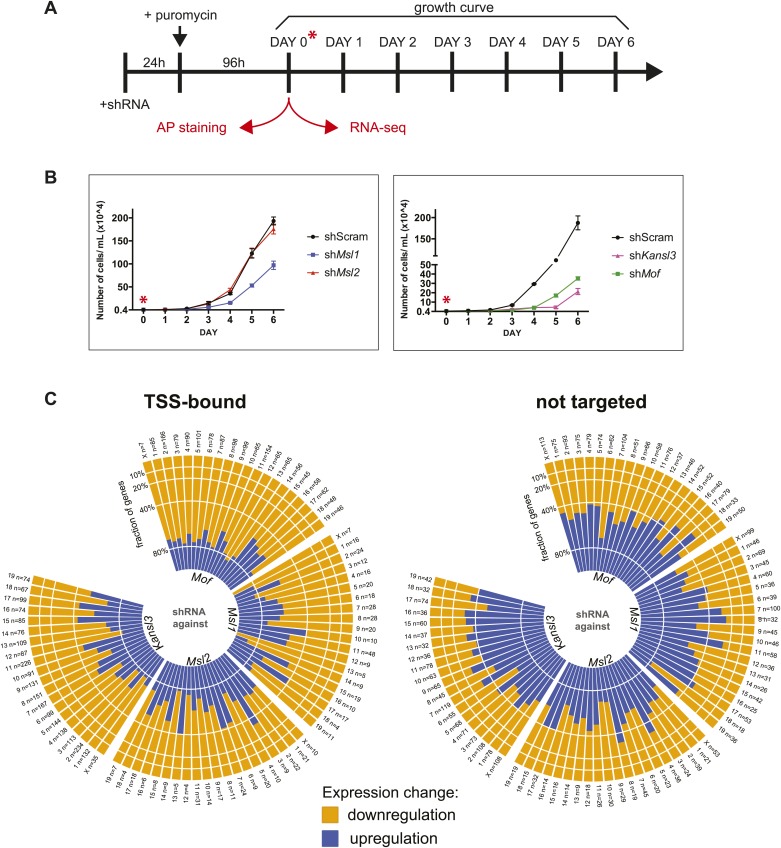
Effects of shRNA-mediated depletion of MOF, MSL1, MSL2 and KANSL3. (**A**) Time course of knockdown experiments. For experimental details see ‘Materials and methods’. Samples for RNA-sequencing and AP staining (Figure 4—figure supplement 4) were extracted 4 days after puromycin selection of shRNA-treated cells. (**B**) Proliferation assay for shRNA-treated cells, starting at day 4 after puromycin selection (Figure 3—figure supplement 3A). (**C**) Bar plots depicting the fractions of genes (per chromosome) that were significantly up- or downregulated in RNA-seq experiments from shRNA-treated cells. The left plot contains genes which were defined as TSS-targets in the respective ChIP-seq samples, the right plot contains genes that were neither classified as TSS- nor as TSS-distal targets. The labels on each bar indicate the chromosome name and the total number of genes that fulfilled the criteria for this chromosome (significantly affected, TSS-bound or non-targeted). See ‘Materials and methods’ for details of the classification. **DOI:** http://dx.doi.org/10.7554/eLife.02024.011

**Figure 3—figure supplement 4.**
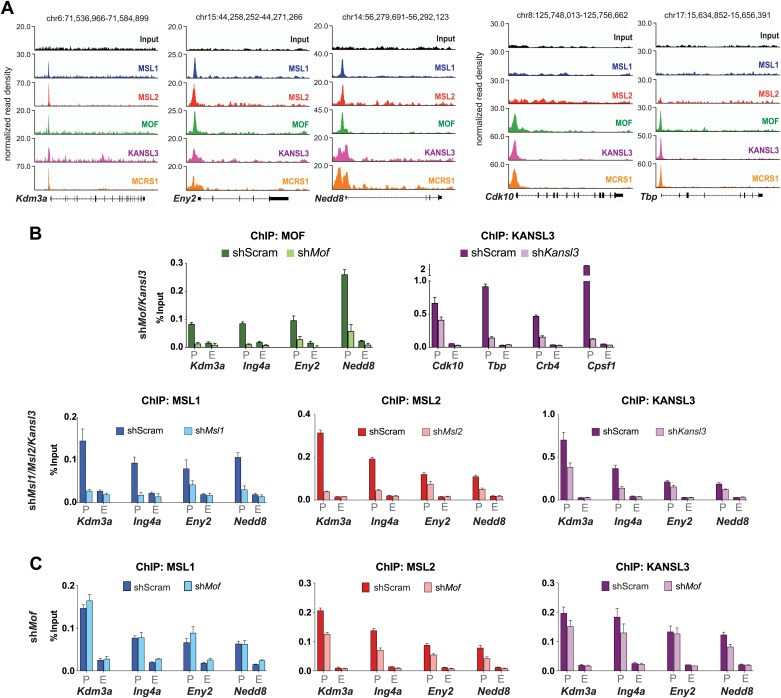
Assessment of ChIP signals around the TSSs of putative target genes as determined by ChIP-seq. (**A**) Genome Browser snapshots of several MSL/NSL (first three from the left) or NSL-only target genes and respective sequencing-depth-normalized ChIP-seq and input signals from ESCs. The exact genomic coordinates are indicated on top of each panel. Gene names are indicated on the bottom. (**B**) ChIP-qPCR validation for MOF (green), KANSL3 (purple), MSL1 (blue) and MSL2 (red) signals. Immunoprecipitated DNA was amplified by qPCR with primer sets positioned at the promoter (P) and end (E) of the coding sequence (Supplementary file 3A). Results are expressed as mean ± SD of three biological replicates; cells were harvested for experiments on day 4 (*Kansl3, Msl1, Msl2*) or 5 (*Mof*) of knockdown. (**C**) ChIP-qPCR for MSL1 (blue), MSL2 (red) and KANSL3 (purple) in ESCs treated with sh-RNA (scrambled or sh*Mof*). Signals on genes were evaluated using primers at the promoter (P), and end (E) of the coding sequence. Results are expressed as mean ± SD of three biological replicates; cells were harvested for experiments on day 5 of *Mof* knockdown. **DOI:** http://dx.doi.org/10.7554/eLife.02024.012

### The TSS-binding of the mouse NSL complex resembles that of the NSL complex in *D. melanogster*

MOF has traditionally been associated with a widespread enrichment along male X-linked genes in flies that is dependent on the MSL proteins (Figure 3D, Figure 3—figure supplement 2A). In our mammalian profiles, despite the presence of the MSLs, we could neither detect X-specific enrichments of MOF nor broad domains of binding along gene bodies. Furthermore, promoter-distal binding sites consisted of narrow peaks and no evidence of spreading from intronic or intergenic regions was observed (Figures 2, 3A,D).

We then examined whether there was a correlation between NSL complex binding in *D. melanogaster* and mouse cells. Indeed, we found that mouse genes that were homologous to NSL complex targets in *D. melanogaster* had a high probability of being bound by the murine NSL complex as well (Pearson's Chi squared test of independence between NSL binding in the fly and the mouse, p-value <2.2e−16). We additionally observed that mouse genes expressed in ESCs and NPCs, whose fly homologues were NSL targets, showed stronger signals for H3K4me3, MOF, KANSL3, and MCRS1 (but not for MSL1 or MSL2) than the mouse homologues of non-NSL-bound *D. melanogaster* genes (Figure 3—figure supplement 2B; lists of NSL-bound and NSL-non-bound fly genes were from Lam et al., 2012). These findings support the notion that the function in housekeeping gene regulation by the *D. melanogaster* NSL complex is evolutionary conserved.

### Depletion of MSL and NSL complex members results in genome-wide downregulation of TSS-target genes

To dissect the biological consequences of the gene targeting by the different MSL and NSL proteins in ESCs, we systematically depleted core members of both complexes (MOF, KANSL3, MSL1, MSL2) (Figure 3—figure supplement 3A). Interestingly, MOF- or KANSL3-depleted cells showed more severe proliferation defects than MSL1- and MSL2-depleted cells (Figure 3—figure supplement 3B). We subsequently performed RNA-seq experiments from shRNA-treated cells and determined their differential expression against the scrambled control to dissect transcriptional outcomes of the depletions at a global level. We found a striking overlap between the differential expression of MSL1 and MSL2 knockdowns and a higher resemblance of MOF-dependent differential expression to that of KANSL3-depletion (Figure 3E). When we specifically focused on genes that we had identified as TSS-bound in our ChIP-seq samples, we found that their transcripts tended to be downregulated in all four knockdowns in comparison to untargeted genes which showed higher fractions of upregulation. These effects were independent of the wild-type expression status of the gene or the chromosome (Figure 3F, Figure 3—figure supplement 3C).

### TSS-binding of MSL1 and KANSL3 does not require MOF

Turning to the assessment of protein levels in shRNA-treated cells, we detected markedly reduced bulk H4K16 acetylation in MSL1- and MOF-depleted cells and only slight reduction upon KANSL3-depletion. This is consistent with previous reports that indicate MSL1 as the major enhancer of MOF's H4K16 acetylation (Kadlec et al., 2011) and demonstrate relaxed substrate specificity for the NSL complex (Zhao et al., 2013b). In addition, we found that MSL1-depletion affected the levels of MSL2 but not of NSL complex members while the depletion of KANSL3 moderately decreased protein levels for both complexes (Figure 3G). ChIP-qPCR assays in MOF-depleted cells revealed that MSL1 and KANSL3 do not require the presence of MOF to bind to gene promoters, which is in agreement with previous observations in *D. melanogaster* (Hallacli et al., 2012; Figure 3—figure supplement 4C).

In summary, our TSS-focused analysis shows that the localized binding of the NSL complex to the promoters of housekeeping genes appears to be a conserved feature between the mammalian and *Drosophila* systems. Unlike in the fly, we do not detect an MSL- and X-chromosome-specific binding mode of MOF in the mouse cell lines. Instead, both complexes narrowly bind to TSSs where their co-occurrence is associated with significantly higher median expression values than those solely bound by the NSL complex. Moreover, we found that MOF is dispensable for the TSS recruitment of its interaction partners and that depletions of the individual proteins predominantly result in the downregulation of TSS-bound genes, further supporting the fact that the promoter-binding of the MSL and the NSL complex is associated with active transcription.

### MSL and NSL complex members individually bind to active enhancers in ESCs

**Figure 4.**
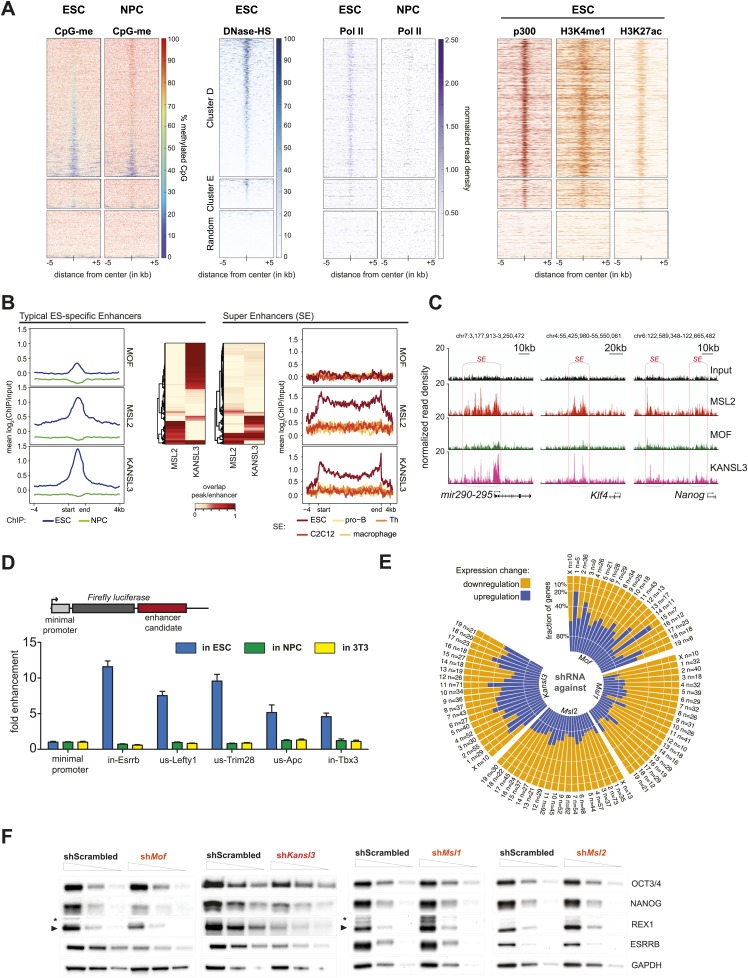
MSL and NSL complex members are enriched at regions with enhancer marks in ESCs. (**A**) Shown here are the fractions of methylated cytosines and ChIP-seq read densities of enhancer markers for regions of ESC-specific enrichments of our proteins of interest. We downloaded the different data from public repositories (see Supplementary file 3A for details) and calculated the values for the regions of the ESC-specific clusters **D** and **E** and random genomic loci. Most data sets used here were from mouse ESC except one RNA Polymerase II (Pol II) sample from NPC. All heatmaps were sorted according to the DNase hypersensitivity values except for CpG methylation heatmaps which were sorted according to their own values. (**B**) Summary plots of input-normalized ChIP-seq signals along typical (TE) and super enhancers (SE) (Whyte et al., 2013). Note that we show the ESC-specific TE only while on the right-hand side we show the signal for SE regions from several cell types. Enhancer regions were scaled to 30 kb (SE) and circa 700 bp (TE). The heatmaps between the summary plots depict how much of each enhancer region overlaps with ChIP-seq peaks of MSL2 or KANSL3. ESC = embryonic stem cells (n = 232), pro-B = progenitor B cells (n = 396), Th = T helper cells (n = 437), C2C12 = myotube cells (n = 536). (**C**) Exemplary genome browser snapshots of annotated super enhancers (SE, pink boxes) for three pluripotency factors displaying the sequencing-depth normalized ESC ChIP-seq signals of MSL2, MOF and KANSL3. See Figure 4—figure supplement 4C for additional examples. (**D**) Luciferase assays demonstrate the biological activity of regions bound by MOF-associated proteins in ESCs (‘in’ stands for intronic region, ‘us’ indicates that the cloned region is upstream of the gene). The firefly luciferase gene was cloned under a minimal promoter together with the putative enhancer region in ESCs, NPCs, and 3T3 cells. The graphs represent at least three independent experiments performed in technical triplicates; error bars represent SEM. (**E**) Bar plots depicting the fraction of significantly up- and downregulated genes per chromosome in the different shRNA-treated cells compared to shScrambled controls (total number of significantly affected genes per sample and chromosome labels are indicated). All genes counted here were classified as TSS-distal target genes in the respective ChIP-seq experiments. See ‘Materials and methods’ for details of the classifications. (**F**) Western blot analyses of the pluripotency factors in scrambled-, *Mof*-, *Kansl3*-, *Msl1*-, and *Msl2*-shRNA-treated male ESCs. For additional analyses in female ESCs see Figure 6C. The respective dilution (100%, 30%, 10%) of loaded RIPA extract is indicated above each panel. Asterisks mark the position of unspecific bands; triangles indicate the protein of interest. GAPDH was used as the loading control. For antibodies see ‘Materials and methods’. **DOI:** http://dx.doi.org/10.7554/eLife.02024.013

In addition to promoter-proximal binding, where both the MSL and NSL complex tend to (co-)occur constitutively in ESCs and NPCs, we identified a large proportion of binding sites where the proteins were present in a dynamic fashion, that is their binding was observed only in ESCs but not in NPCs (Figure 2, clusters D and E). In contrast to the binding mode represented by clusters A and B (Figure 2), here MSL2, MCRS1, and KANSL3 were predominantly enriched within introns and intergenic regions that underwent significant CpG methylation upon differentiation (e.g., from median 50% CpG methylation in ESCs to more than 80% in NPCs for cluster D; bisulfite sequencing data from Stadler et al., 2011). As shown in Figure 4A, CpG methylation in NPCs was particularly pronounced around the center of the regions with significant ChIP enrichments in ESCs, indicating a correlation between the loss of ChIP-seq signal for MOF, MSL1, MSL2, KANSL3 and MCRS1, and DNA methylation upon differentiation. In addition, the regions of cluster D and, to a lesser extent the MSL1-rich cluster E (Figure 2), showed highly localized enrichments of DNase hypersensitivity sites (DNase HS), RNA Polymerase II (Pol II), p300, methylation of histone 3 on lysine 4 (H3K4me1), and acetylation of histone 3 on lysine 27 (H3K27ac) in ESCs (Figure 4A), which are characteristic features of enhancer regions. We thus examined whether MOF and its interaction partners were enriched on known enhancer regions, using lists of typical and super enhancers defined by binding sites of the pluripotency factors SOX2, NANOG, and OCT4 (Whyte et al., 2013), as well as sets of active and poised enhancers based on histone mark signatures (Creyghton et al., 2010).

**Figure 4—figure supplement 1.**
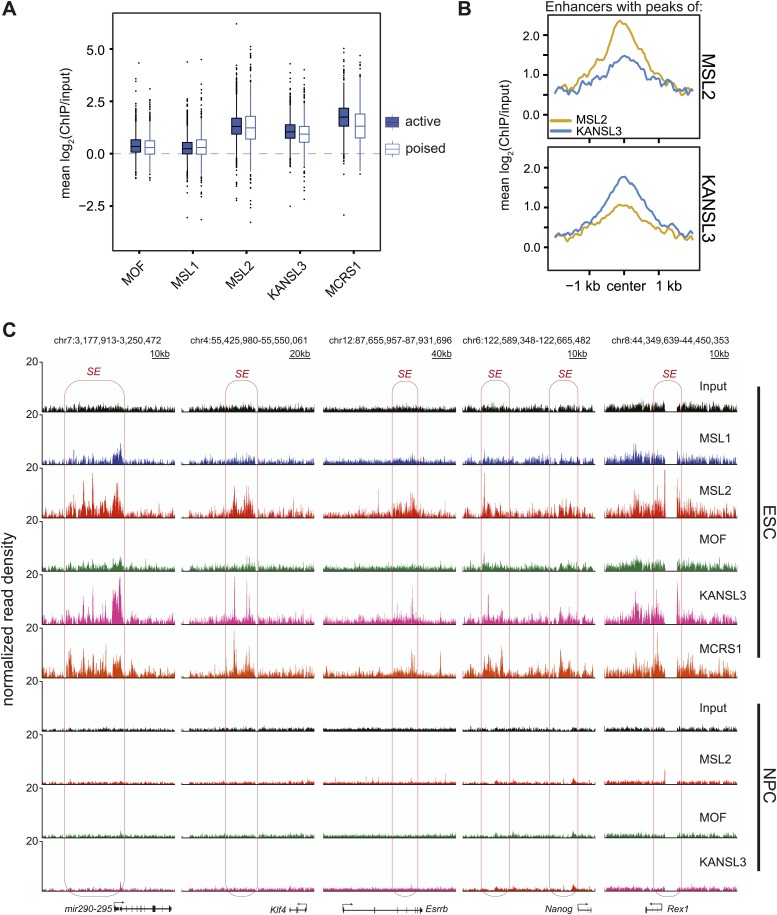
MSL2 and KANSL3 show strong enrichments at typical and super enhancers in ESCs. (**A**) Boxplots demonstrating the distribution of mean ChIP enrichments for enhancer regions defined by H3K4me1 and H3K27ac marks in ESCs (see Creyghton et al., 2010 for details) that overlap with the clusters of binding defined by our ChIP-seq samples. Mean values were extracting using the UCSCtool bigWigAverageOverBed. (**B**) Summary plots for typical enhancer regions (Whyte et al., 2013) that overlapped with either MSL2 (top) or KANSL3 (bottom) peaks. Different colors indicate different ChIP-seq signals. Related to the heatmaps of Figure 4B. (**C**) Genome browser snapshots of sequencing-depth normalized ChIP-seq and input profiles for super enhancers of key pluripotency factors. **DOI:** http://dx.doi.org/10.7554/eLife.02024.014

**Figure 4—figure supplement 2.**
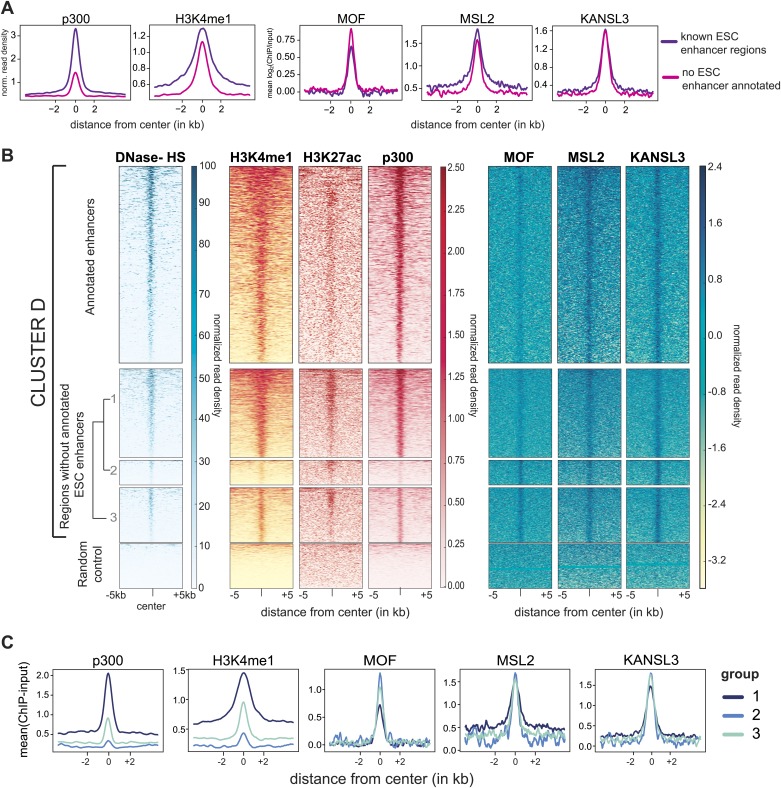
MOF is moderately enriched at non-canonical enhancers. (**A**) Summary plots of ChIP-seq values for binding sites belonging to cluster D. The regions were divided based on the presence or absence of annotated ESC enhancers (Creyghton et al., 2010; Whyte et al., 2013). (**B**) Heatmaps of ChIP-seq read densities of known enhancer markers for the ESC-specific binding sites of our proteins of interest (cluster D, see Figure 2) and random genomic regions. The binding sites of cluster D (excluding regions with TSSs) were divided into two basic groups based on the presence or absence of known ESC enhancers (Creyghton et al., 2010; Whyte et al., 2013). The latter group was further divided into three (arbitrarily numbered) sub-clusters based on hierarchical clustering of the values from DNase hypersensitivity sites, p300, H3K4me1 and our MOF sample (in ESCs). Heatmaps of the ESC-enhancer-containing regions were sorted according to p300, those of the sub-clustered regions were sorted according to the MOF signal. (**C**) Related to (**B**), shown here are the corresponding summary plots of ChIP-seq values for cluster D binding sites that do not overlap with annotated enhancer regions (bottom part of the heatmaps in the figure above). The three indicated groups are based on the hierarchical clustering that was performed on p300, H3K4me1 and MOF values (‘Regions without annotated ESC enhancers’ in (**B**)). **DOI:** http://dx.doi.org/10.7554/eLife.02024.015

**Figure 4—figure supplement 3.**
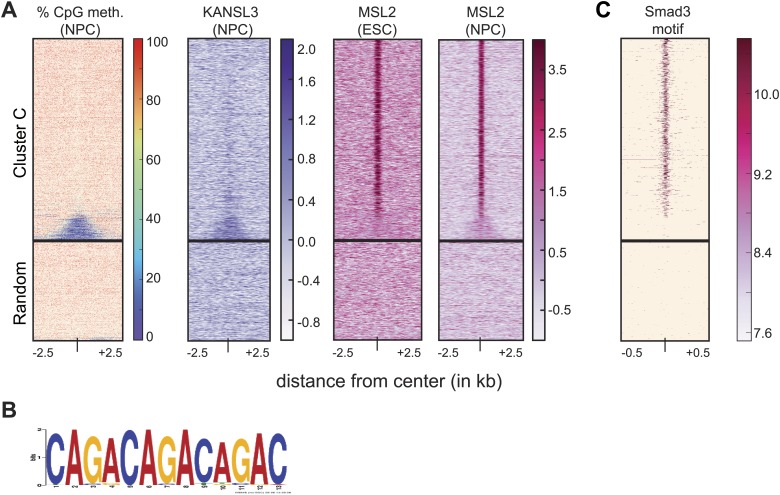
MSL2 has intergenic binding sites in DNA-hypomethylated regions that are enriched for SMAD3 binding sites. (**A**) We extracted the percentage of methylated CpGs and the input-normalized ChIP-seq values from KANSL3 and MSL2 and 5 kb surrounding the center of the regions belonging to cluster C (Figure 2) and random genomic control regions. All heatmaps were sorted according to the percentages of methylated CpGs (Stadler et al., 2011). (**B**) Motif obtained by MEME analysis on the top 200 MSL2 peaks within cluster **C**. (**C**) Same as for (**A**), except that the score was the motif hit score for SMAD3 for 1 kb. See ‘Materials and methods’ for details. **DOI:** http://dx.doi.org/10.7554/eLife.02024.016

**Figure 4—figure supplement 4.**
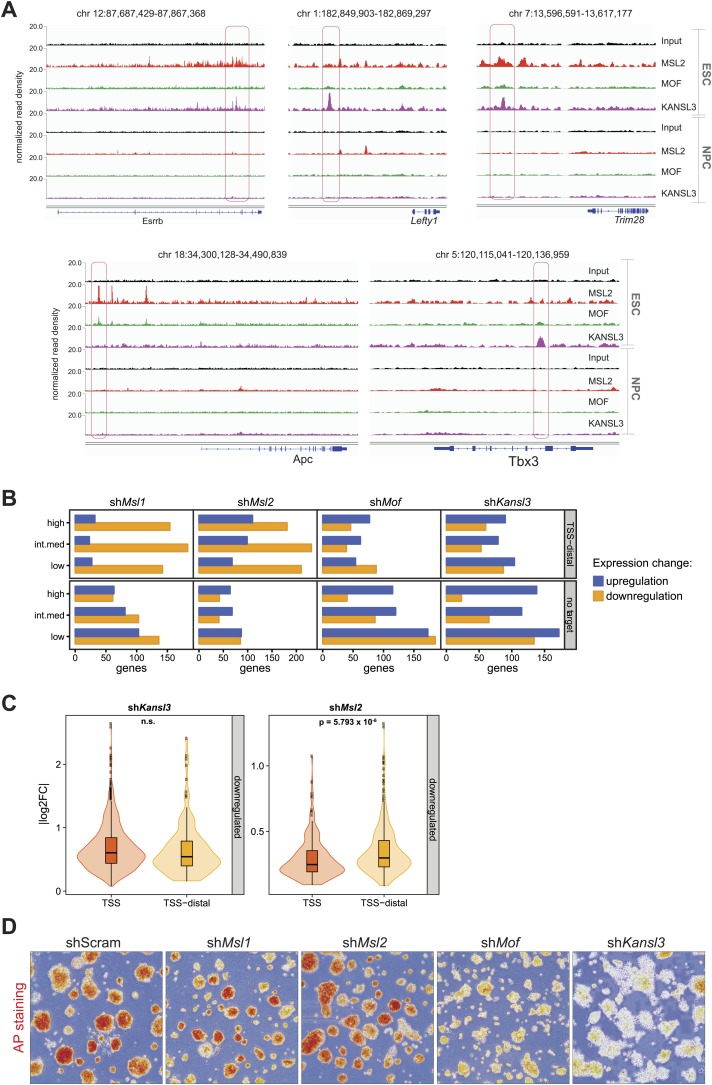
Biological significance of the TSS-distal binding sites of the investigated proteins. (**A**) Genome browser snapshots of sequencing-depth normalized ChIP-seq and input profiles. Pink boxes mark the regions cloned and transfected into ESCs and NPCs for luciferase assays (Figure 4D). (**B**) Genes that were significantly up- or downregulated in the respective shRNA-treatments compared to shScrambled were classified according to ChIP-seq peak overlaps (TSS-distal, no target) and expression strength in wild-type ESCs (high, intermediate, low). See ‘Materials and methods’ for details of the classifications. (**C**) Distribution of absolute log_2_ fold changes (sh*Kansl3* or sh*Msl2* compared to shScrambled) for significantly downregulated genes. Different shades of orange indicate different target classes based on ChIP-seq experiments for KANSL3 or MSL2, respectively. p-values were calculated with Welch *t* test. (**D**) Alkaline phosphatase staining and morphology of ESC colonies in indicated knockdowns after 4 days growth under puromycin selection (Figure 3—figure supplement 3A). MOF- and KANSL3-depleted cells demonstrate reduced alkaline phosphatase positive colonies with increased differentiation compared with MSL1- and MSL2-depleted cells and scrambled control. **DOI:** http://dx.doi.org/10.7554/eLife.02024.017

Interestingly, MSL2, KANSL3 and MCRS1, but not MOF and MSL1, showed profound enrichments for active and poised ESC enhancers (Figure 4—figure supplement 1A) as well as along the regions of super enhancers that have been described as being particularly important for maintenance of cell identity (Whyte et al., 2013).

The signals of MSL2 and KANSL3 were specific for ESC enhancers and wide-spread along super enhancer regions (Figure 4B,C). We noted that enhancers overlapping with MSL2 ChIP-seq peaks tended to show lower KANSL3 enrichments and vice versa, implying that MSL2 and KANSL3 preferred different enhancer regions (heatmaps in Figure 4B, Figure 4—figure supplement 1B). MOF was not enriched at super enhancers and generally, its binding to TSS-distal sites was much less pronounced than to gene promoters (Figure 4—figure supplement 1A, 1C, Figure 2). Like for TSS-specific binding, MOF was not alone (87% of TSS-distal MOF peak regions overlapped with either KANSL3 or MSL2). Since a recent report showed H4K16 acetylation to be present at p300- and H3K27-acetylation-independent enhancer regions (Taylor et al., 2013), we analyzed the moderate TSS-distal enrichments of MOF in more detail and observed a slight preference for TSS-distal regions that were not overlapping with previously published ESC enhancer regions (Figure 4—figure supplement 2A). In fact, we detected the strongest MOF signals in regions with rather low enrichments of known enhancer marks (see DNase HS, p300, H3K4me1, H3K27ac in Figure 4—figure supplement 2B and 2C), which suggested a preferred binding of MOF outside canonical ESC regulatory regions.

In addition to ESC-specific binding of MSL2 and KANSL3 to predicted enhancers, we also identified a very distinct set of TSS-distal binding sites by MSL2 to introns and intergenic regions without enhancer-associated marks (cluster C in Figure 2). Approximately, 81% of these cluster C regions had solitary MSL2 enrichments without significant signals of any of the other ChIPed proteins. Interestingly, these MSL2 binding sites increased in number and binding strength upon differentiation to NPCs (829 solitary MSL2 peaks in ESCs compared to 3635 in NPCs). In contrast to the previously described binding sites that were characterized by the prevalence of open, active chromatin (Figures 3 and 4), here MSL2 was excluded from hypo-methylated DNA regions (Figure 4—figure supplement 3A; note the different behavior of KANSL3). When we searched the unique MSL2 binding sites for DNA motifs, we obtained a (CAGA)_n_ motif (Figure 4—figure supplement 3B) that was previously described as a binding site for SMAD3, a transcription factor that translates the TGF-beta receptor response into gene expression regulation (Zawel et al., 1998). When we subsequently scanned all the binding sites for the presence of the published, original SMAD3 motif, we found a strikingly specific signal for the center of the solitary MSL2 ChIP-seq peaks (Figure 4—figure supplement 3C).

We conclude that MOF, MSL2 and KANSL3 specifically recognize ESC enhancers. In contrast to MSL-MOF-NSL co-occurrence at housekeeping gene promoters, we found evidence for differential and independent binding of the individual proteins to gene bodies and intergenic regions suggesting the potential for distinct tissue-specific regulatory functions of MSL2 and KANSL3. These data reveal a newly evolved function of MSL2 and KANSL3 in mammals, which has not been observed in flies.

### Genes associated with TSS-distal binding sites of MSL1 and MSL2 are frequently downregulated in cells lacking MSL1 or MSL2

To study the functional implications of the binding of MSL2 and KANSL3 to putative ESC enhancers, we first tested five different regions located near genes related to pluripotency and self-renewal (Hu et al., 2009; Young, 2011). Using luciferase reporter constructs, we found strong transcriptional enhancement for all tested regions in ESCs, but not in NPCs or 3T3 cells which correlated with the presence of MSL2 and/or KANSL3 and MCRS1 in ESCs only (Figure 4D, Figure 4—figure supplement 4A).

We then used our RNA-seq data sets from MSL1-, MSL2-, MOF-, and KANSL3-depleted cells to assess the effects on the transcription of those genes that were not bound at promoters, but had been predicted by GREAT (McLean et al., 2010) to be regulated by TSS-distal binding sites of the respective protein. As shown in Figure 4E, we again found similar effects for KANSL3- and MOF-depleted cells compared to MSL1- and MSL2-depleted cells with the latter group showing genome-wide downregulation of predicted target genes. In fact, the numbers of TSS-distal targets of MSL1 or MSL2 that were significantly reduced in the respective shRNA-treatments were markedly larger than for genes where MSL1 or MSL2 bound to the promoter (compare Figure 3F with Figure 4—figure supplement 4B). Moreover, in MSL2-, but not KANSL3-depleted cells, the effects on TSS-distally targeted genes were slightly stronger than for TSS-targets (Figure 4—figure supplement 4C).

### Depletions of MOF and KANSL3, but not of MSL complex members affect key pluripotency factors

While TSS-binding predominantly occurred at housekeeping genes, we noticed that the majority of enhancer regions associated with key pluripotency factors (e.g., SOX2, ESRRB, MYC, REX1, TBX3, NANOG) were strongly enriched for MSL2 and KANSL3. We thus assessed the effects of the protein depletions on pluripotency factors in ESCs and found strongly reduced levels of NANOG, REX1, and ESRRB in MOF- or KANSL3-depleted cells. Surprisingly, the pluripotency factors remained almost unaffected in cells depleted of MSL1 or MSL2 (Figure 4F). These contrasting results were mirrored by decreased levels of alkaline phosphatase (AP) in MOF- and KANSL3-, but not in MSL1- or MSL2-depleted cells (Figure 4—figure supplement 4D).

These findings indicate that despite their frequent effects on TSS-distally targeted genes, MSL1 and MSL2 might not show dominant effects at genes that are bound by KANSL3 as well. Therefore, we specifically searched for regions without KANSL3 binding to identify putative MSL-specific functions.

### The MSL complex binds multiple loci within the X inactivation center

As described previously, we identified only a small subset of regions in the mouse genome where MSL complex members were enriched exclusively (see cluster E in Figure 2). Strikingly, several of these binding sites fall into a region known as the X inactivation center (XIC). The XIC is the X-chromosomal region necessary and sufficient to control the inactivation of one of the two X chromosomes in females (reviewed in Pollex and Heard, 2012).

**Figure 5.**
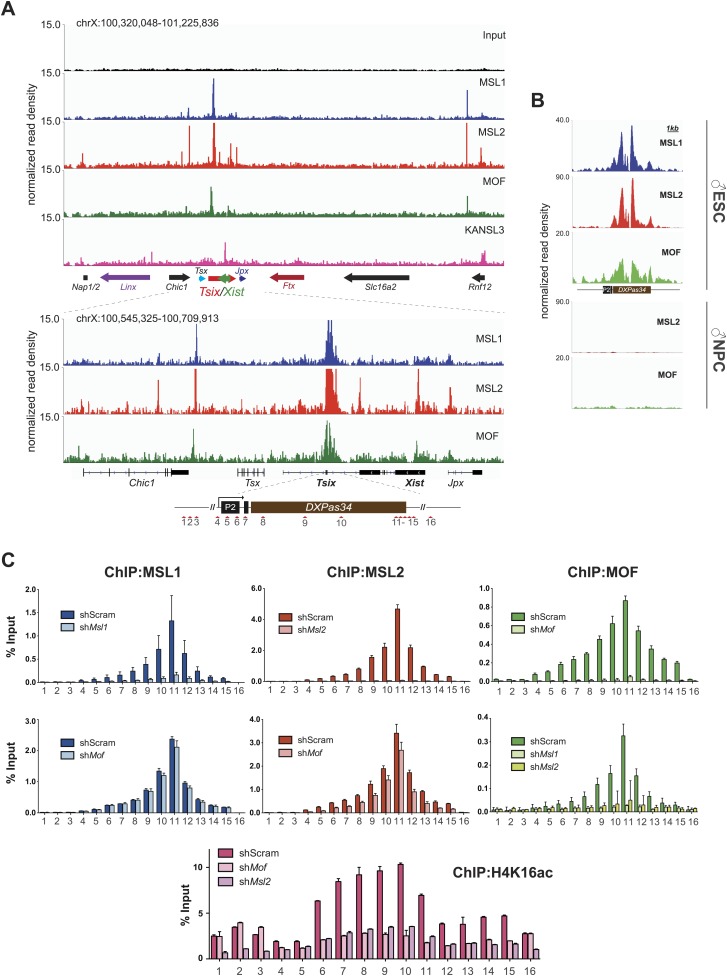
The MSL complex binds multiple loci within the X inactivation center including the *Tsix DXPas34* minisatellite enhancer. (**A**) Genome browser snapshots of the mouse X inactivation center (approximately 0.9 Mb) (upper panel) plus enlargement of the 164 kb region between *Chic1* and *Jpx/Enox* (lower panel). The signals shown are the sequencing-depth normalized profiles for ChIP-seq from ESCs (for corresponding profiles in NPCs see Figure 5—figure supplement 1A); colored arrows indicate genes of lncRNAs. The schematic representation of the *DXPas34* locus depicts the locations of the primer pairs that were used for ChIP-qPCR analyses (Supplementary file 3B). (**B**) Genome browser snapshots of the *DXPas34* minisatellite of sequencing-depth normalized ChIP-seq profiles in ESCs and NPCs. (**C**) ChIP-qPCR analyses of MSL1 (blue), MSL2 (red), MOF (green), and H4K16 acetylation (purple) across the *Tsix* major promoter (*P2*) and the *DXPas34* enhancer in male ESCs treated with the indicated shRNAs. For corresponding ChIP-qPCR in female ESCs see Figure 5—figure supplement 1C. Panels in the middle show the effects of MOF depletion on the recruitment of MSL1 and MSL2 to *DXPas34* and vice versa. The bottom panel shows effects of depletion of control (dark pink), MOF (light pink) and MSL2 (purple) on the H4K16 acetylation signal. The labels of the x axes correspond to the arrowheads in (**A**). Results are expressed as mean ± SD of three biological replicates; cells were harvested on day 4 (*Msl1*, *Msl2*) or 5 (*Mof*) after shRNA treatment. For primer pairs see Supplementary file 3C. **DOI:** http://dx.doi.org/10.7554/eLife.02024.018

The XIC site with the strongest concomitant enrichments of MSL1, MSL2 and MOF was the major promoter (P2) of *Tsix* and its intronic minisatellite—*DXPas34* (Figure 5A,B). *DXPas34* is a well-characterized tandem repeat that serves as a binding platform for multiple transcription factors and contains bidirectional enhancing properties essential for the expression of *Tsix*, the antisense transcript of *Xist* (Debrand et al., 1999; Cohen et al., 2007; Donohoe et al., 2007; Navarro et al., 2010; Gontan et al., 2012). In rodents, *Tsix* antisense transcription across the *Xist* promoter is required for regulating the levels of *Xist* accumulation. In turn, *DXPas34* deletion impairs the recruitment of Pol II and TFIIB to the major promoter of *Tsix* causing its downregulation (Vigneau et al., 2006).

**Figure 5—figure supplement 1.**
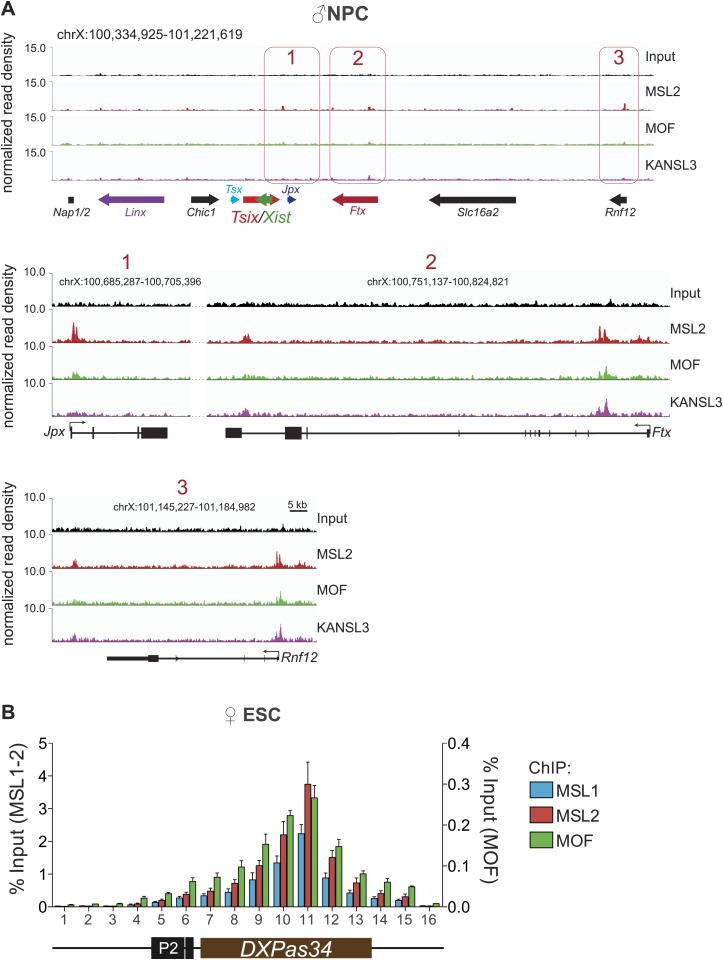
The MSL proteins bind to multiple loci within the X inactivation center (XIC). (**A**) Genome browser snapshots of the mouse XIC (top panel) with three enlargements on *Jpx*, *Ftx* and Rnf12 genes (lower panels). Red boxes with corresponding numbers mark the enlarged regions presented in the lower panels. The exact genomic coordinates are indicated on top of each panel, arrows represent genes. The signals shown are the sequencing-depth normalized ChIP-seq profiles in NPCs. (**B**) ChIP analysis of MSL1, MSL2 and MOF across the *DXPas34* minisatellite in female ESCs. The x-axis labels indicate the genomic coordinates corresponding to the arrowheads in Figure 5A. The y-axes show the percentage of ChIP recovery for MSL1 and MSL2 (left-hand side) and MOF (right-hand side) normalized to input. For all ChIP experiments, three biological replicates were used; all results are expressed as mean ± SD. **DOI:** http://dx.doi.org/10.7554/eLife.02024.019

In addition to the *DXPas34* binding site, we detected MSL peaks on the promoters, gene bodies and intronic regions of other key XIC regulators including the genes of the long non-coding (lnc) RNAs *Xist* and *Jpx*. Additionally, we observed peaks upstream of the *Tsx* gene and both at the TSS and downstream of the *Rnf12* gene (Figure 5A). Products of all of these genes were shown to play important roles in orchestrating the process of X inactivation (Stavropoulos et al., 2001; Shin et al., 2010; Tian et al., 2010; Anguera et al., 2011; Chureau et al., 2011; Gontan et al., 2012; Sun et al., 2013).

The XIC binding of MSL-MOF was specific to ESCs, as almost all enrichments were abolished upon differentiation, except for some loci upstream of *Xist* where traces of binding could still be detected in NPCs (e.g., *Ftx* and *Jpx* TSS, Figure 5—figure supplement 1A).

We next confirmed the high ChIP-seq enrichments of MSL1, MSL2 and MOF and assessed H4K16 acetylation on the major promoter of *Tsix* and along *DXPas34* with ChIP-qPCR assays covering the entire region in male and female ESCs (Figure 5C, Figure 5—figure supplement 1B). Interestingly, the recruitment of MOF was almost completely abolished in both MSL1- and MSL2-depleted cells, whereas the depletion of MOF had no effect on MSL1 and MSL2 binding to the *Tsix* major promoter and *DXPas34* (Figure 5C). H4K16 acetylation ChIP signals were severely reduced in both MOF- and MSL2-depleted cells. These results are in agreement with our global observations (Figure 3G, Figure 3—figure supplement 4C) and indicate that MSL1 and MSL2 are together necessary and sufficient for the recruitment of MOF and for the deposition of H4K16 acetylation at *DXPas34*.

### MSL1 and MSL2 are important for *Tsix* expression

**Figure 6.**
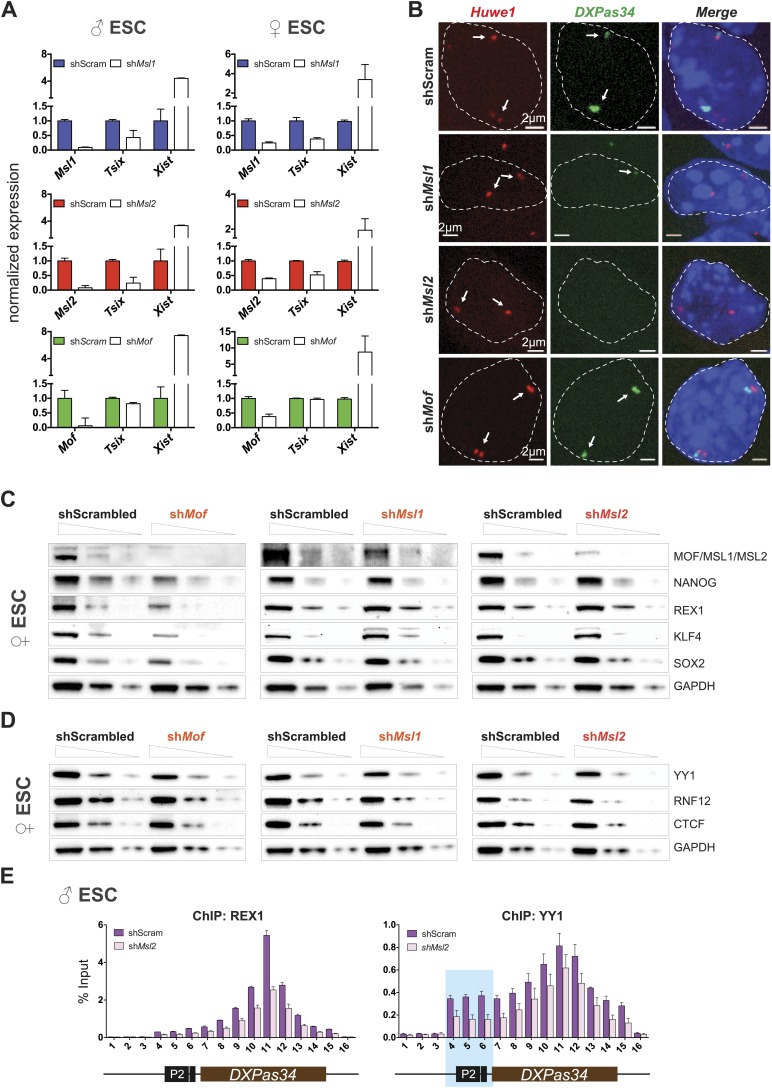
Depletion of MSL1 and MSL2 leads to downregulation of *Tsix* with concomitant upregulation of *Xist*. (**A**) Gene expression analysis for the indicated genes in male and female ESCs treated with scrambled RNA (shScram) or shRNA against *Msl1*, *Msl2,* or *Mof*. All results are represented as relative values normalized to expression levels in shScram (normalized to *Hprt*) and expressed as means ± SD in three biological replicates. (**B**) RNA-FISH for *Huwe1* (red) and *DXPas34* (green) in: scrambled control, sh*Msl1*-, sh*Msl2*-, and sh*Mof*-treated female ESCs. Nuclei were counterstained with DAPI (blue). White arrows denote foci corresponding to *Huwe1* or *Tsix*; dashed lines indicate nuclei borders. For additional images, phenotypes and quantifications see Figure 6—figure supplement 1A–C. For probe references see ‘Materials and methods’. (**C**) Western blot analyses of the pluripotency factors in scrambled-, *Mof*-, *Msl1*-, and *Msl2*-shRNA-treated female ESCs. For corresponding expression analyses see Figure 6—figure supplement 1D,E. The respective dilution (100%, 30%, 10%) of loaded RIPA extracts is shown above each panel. GAPDH was used as the loading control. For antibodies see ‘Materials and methods’. (**D**) Western blot analyses of the transcription factors involved in regulation of the XIC in scrambled-, *Mof*-, *Msl1-,* and *Msl2*-shRNA-treated female ESCs. The respective dilution (100%, 30%, 10%) of loaded RIPA extracts is shown above each panel. GAPDH was used as the loading control. (**E**) ChIP-qPCR analysis of REX1 (left panel) and YY1 (right panel) across the *Tsix* major promoter (P2) and *DXPas34* in male ESCs treated with the indicated shRNAs. The labels of the x axes correspond to the arrowheads in Figure 5A. For all ChIP experiments, three biological replicates were used; results are expressed as mean ± SD; cells were harvested on day 4 (*Msl2*) or 5 (*Mof*) after shRNA treatment. **DOI:** http://dx.doi.org/10.7554/eLife.02024.020

To directly assess the functional outcome of MOF-, MSL1-, and MSL2-depletions, we studied the expression of *Tsix* and *Xist* in shRNA-treated ESCs. Unexpectedly, only MSL1- and MSL2-, but not MOF-depletion led to pronounced downregulation of *Tsix* both in male and female ESCs (Figure 6A; note that in our RNA-seq data set for MSL2-depleted cells, *Tsix* was among the five most strongly downregulated genes). Downregulation of *Tsix* was accompanied by moderately elevated *Xist* RNA levels in MSL1- and MSL2-depleted ESCs whereas depletion of MOF yielded the most pronounced (8–15-fold) upregulation of *Xist* without affecting *Tsix*.

**Figure 6—figure supplement 1.**
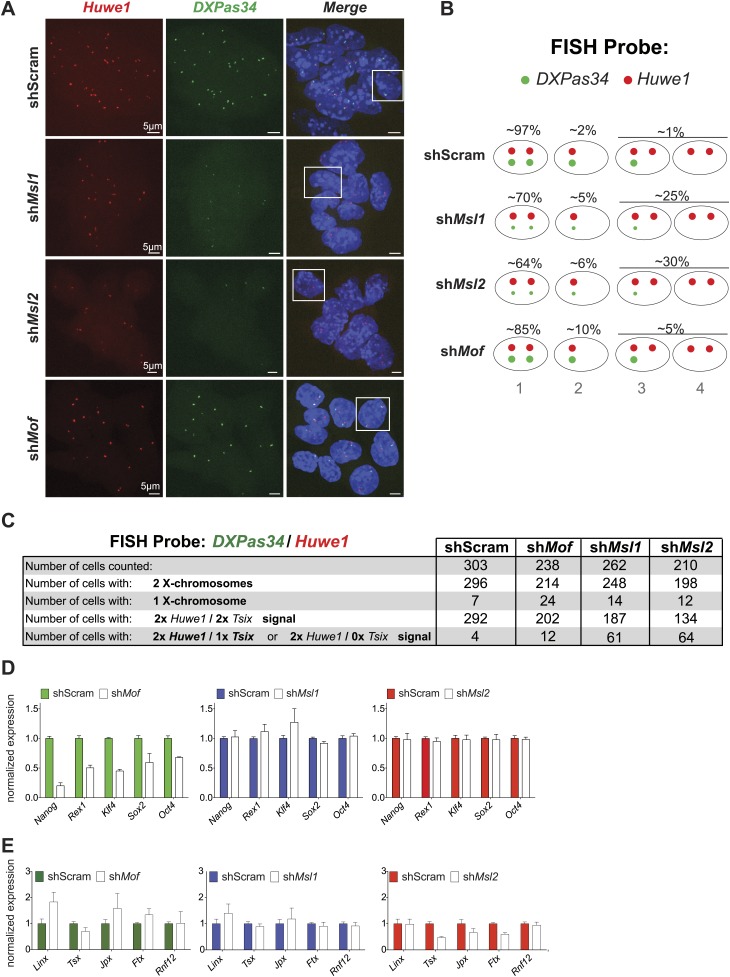
Cells depleted of MSL1 or MSL2, but not MOF show loss of *DXPas34* foci. (**A**) RNA-FISH for *Huwe1* (red) and *DXPas34* (green) in shScrambled-, sh*Msl1*-, sh*Msl2*-, and sh*Mof*-treated female ESCs. Shown here are examples of RNA-FISH signals for multicellular colonies and loss of *DXPas34* signal in MSL1- and MSL2-depleted cells. White boxes indicate cells enlarged and resented in Figure 6B. For all experiments, nuclei were counterstained with DAPI (blue). (**B**) Summary of RNA-FISH for *DXPas34* and *Huwe1*. Red dots indicate the number of X chromosomes and green dots, *DXPas34* foci (smaller dot = reduced signal). Phenotypes that we observed in knockdowns are categorized into four groups containing cells with equal *Huwe1*/*DXPas34* ratio and with *DXPas34* loss. The percentages indicate how many cells per population showed the respective phenotype. (**C**) Corresponding to Figure 6B. Summary of total cell counts from RNA-FISH for (*DXPas34*) and *Huwe1* in MSL1-, MSL2-, or MOF-depleted female ESCs. (**D**) Gene expression analysis for the indicated genes in female ESCs treated with scrambled RNA (shScram) or shRNA against *Mof*, *Msl1* and *Msl2*. All results are represented as relative values normalized to expression levels in shScram (normalized to *Hprt*) and expressed as means ± SD in three biological replicates. (**E**) Gene expression analysis for genes of the XIC in female ESCs treated with scrambled RNA or shRNA against *Msl1*, *Msl2* or *Mof*. All results are represented as relative values normalized to expression levels in shScrambled (normalized to Hprt) and expressed as means ± SD for three biological replicates. **DOI:** http://dx.doi.org/10.7554/eLife.02024.021

To determine the effects on *Tsix* in individual cells, we next performed RNA-FISH with probes against *DXPas34* and *Huwe1* in female ESCs (*Huwe1* was used to mark X chromosomes, for probe references see ‘Materials and methods’). The RNA-FISH confirmed the qPCR results as we observed global reduction and in many cases elimination of *DXPas34* signals in MSL1- and MSL2-, but not in MOF-depleted cells (Figure 6B, Figure 6—figure supplement 1A–C).

We next wanted to understand the mechanistic differences between the *Tsix*-specific and the *Tsix*-independent effects on *Xist* levels that we found for depletions of MSL1/MSL2 and MOF, respectively. As pluripotency factors are additional regulators of *Xist* (Navarro et al., 2008; Nesterova et al., 2011), we assessed the consequences of the different knockdowns on the *Xist*-related pluripotency network in female ESCs. Like for MOF- and KANSL3-depletions in male ESCs (Figure 4F), the depletion of MOF (but not of MSL1 or MSL2) in female ESCs resulted in a significant decrease of transcript and protein levels of pluripotency factors that had previously been associated with *Xist* repression (e.g., NANOG and REX1; see Figure 6C, Figure 6—figure supplement 1D).

Taken together, we detect direct binding of MSL complex members to several loci within the X inactivation center including the *Tsix/Xist* locus. Depletion of MSL1 or MSL2, but not MOF led to severe downregulation of *Tsix* expression while depletion of MOF, MSL1, or MSL2 resulted in elevated *Xist* levels. These results indicate a direct regulatory function of MSL1 and MSL2 on the *DXPas34* locus and an indirect NSL-associated MOF effect on *Xist* expression through the pluripotency network.

### Depletion of MSL1 and MSL2 leads to impaired recruitment of REX1 and YY1 to regulatory regions of *Tsix*

As loss of MSL1 and MSL2 did not affect the core pluripotency network, we set out to explore what might be the impact of MSL depletion on XIC genes (other than *Tsix* and *Xist*) and transcription factors involved in their regulation. As shown in Figure 6—figure supplement 1E, we observed mild effects on the expression of XIC-encoded genes involved in the regulation of X inactivation. Only depletion of MSL2 led to significant downregulation of *Ftx* and *Jpx* genes whose promoters were bound by MSL1 and/or MSL2 (Figure 5A). On the other hand, depletion of MOF led to moderate upregulation of *Linx* lncRNA, which acts synergistically with *Tsix* (Nora et al., 2012).

Neither the depletion of MSL1 and MSL2 nor the depletion of MOF significantly influenced protein levels of RNF12, YY1, or CTCF that are known regulators of the XIC (Figure 6D; Donohoe et al., 2007, 2009; Jonkers et al., 2009; Shin et al., 2010; Jeon and Lee, 2011). Since REX1 and YY1 bind and regulate the *Tsix* locus (Donohoe et al., 2007; Gontan et al., 2012), we subsequently tested whether MSL depletion would affect the recruitment of these factors to the *Tsix* major promoter and *DXPas34*. Indeed, the depletion of MSL2 led to significant reduction of REX1 ChIP signals across the *DXPas34* locus whereas the effect on YY1-targeting was less pronounced and restricted to the *Tsix* major promoter (P2) (Figure 6E).

### Knockdown of *Msl1* and *Msl2* results in enhanced accumulation of *Xist* and X-chromosomal coating in differentiating female ESCs

**Figure 7.**
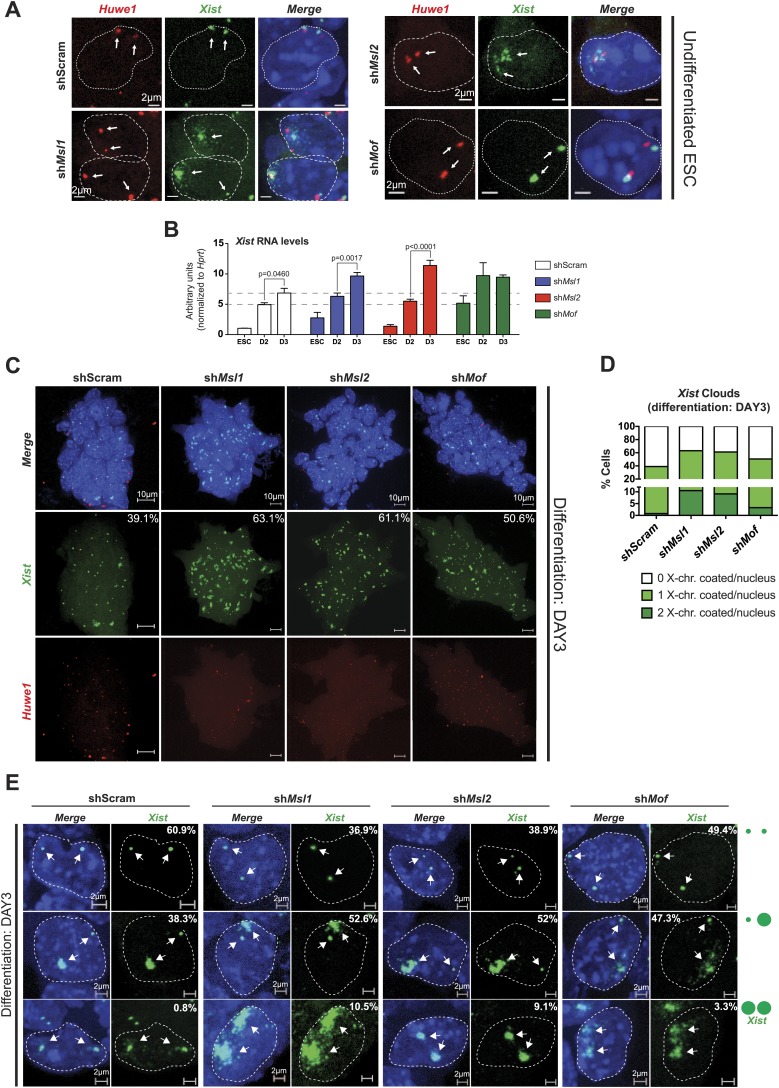
MSL1 and MSL2 depletion leads to enhanced and chaotic *Xist* accumulation in early differentiation. (**A**) RNA-FISH for *Huwe1* (red) and *Xist* (green) in: scrambled control, sh*Msl1*-, sh*Msl2*-, and sh*Mof*-treated female ESCs. Nuclei were counterstained with DAPI (blue). White arrows denote foci corresponding to *Huwe1* or *Xist*; dashed lines indicate nuclei borders. For additional images, phenotypes and quantifications see Figure 7—figure supplement 1B–D. For probe references see ‘Materials and methods’. (**B**) Expression analysis for *Xist* in undifferentiated, day 2 (D2) and day 3 (D3) differentiating female ESCs treated with scrambled RNA (shScram) or shRNA against *Mof*, *Msl1,* and *Msl2*. All results are represented as arbitrary units (*Xist* expression in undifferentiated ESCs = 1) normalized to expression levels in shScram (normalized to *Hprt*) and expressed as means ± SD in three biological replicates. p-values for D2-to-D3 expression change were obtained using unpaired *t* test. (**C**) RNA-FISH for *Huwe1* (red) and *Xist* (green) in: scrambled control, sh*Msl1*-, sh*Msl2*-, and sh*Mof*-treated differentiating female ESCs. Nuclei were counterstained with DAPI (blue). RNA-FISH was performed on the sixth day of knockdown (after 72 hr of differentiation). Percentages indicate number of cells with at least one *Xist* cloud for each of the knockdowns. For additional images of multicellular colonies see Figure 7—figure supplement 2A. (**D**) Bar plot summarizing the percentage of *Xist* clouds for individual knockdowns in differentiating (DAY3) female ESCs for individual knockdowns. Cells were divided into three categories: cells carrying no *Xist* clouds (white), single *Xist* cloud (light green), or two *Xist* clouds (dark green). For quantifications, see Figure 7—figure supplement 2B. (**E**) RNA-FISH for *Xist* (green) in: scrambled control, sh*Msl1*-, sh*Msl2*-, and sh*Mof*-treated differentiating (DAY3) female ESCs. Here, we show examples of individual nuclei carrying different patterns of *Xist* accumulation. Percentages correspond to the frequency of the shown *Xist* pattern within the population of cells. White arrows denote *Xist* foci; dashed lines indicate nuclei borders. For quantifications see Figure 7—figure supplement 2B. **DOI:** http://dx.doi.org/10.7554/eLife.02024.022

We next assessed the consequence of MSL-dependent reduction of *Tsix* levels and concomitant upregulation of *Xist* at a cellular level using RNA-FISH for *Xist* upon depletion of individual MSL complex members (for probe reference see ‘Materials and methods’). Interestingly, we observed accumulating *Xist* lncRNA and X-chromosomal coating in a small fraction of MSL1- and MSL2-depleted female ESCs (but not MOF-depleted cells; 4–5% of the cell population in sh*Msl1* and sh*Msl2* with comparison to 0.5% in scrambled control, see Figure 7A and Figure 7—figure supplement 1A–C). These findings suggest that the MSL1- and MSL2-dependent downregulation of *Tsix* is sufficient to cause occasional accumulation of *Xist* lncRNA in undifferentiated female ESCs. The different outcomes following MOF and MSL1/MSL2 depletion on *Xist* confirmed the notion that MOF and MSL1/MSL2 influence the XIC via different mechanisms.

**Figure 7—figure supplement 1.**
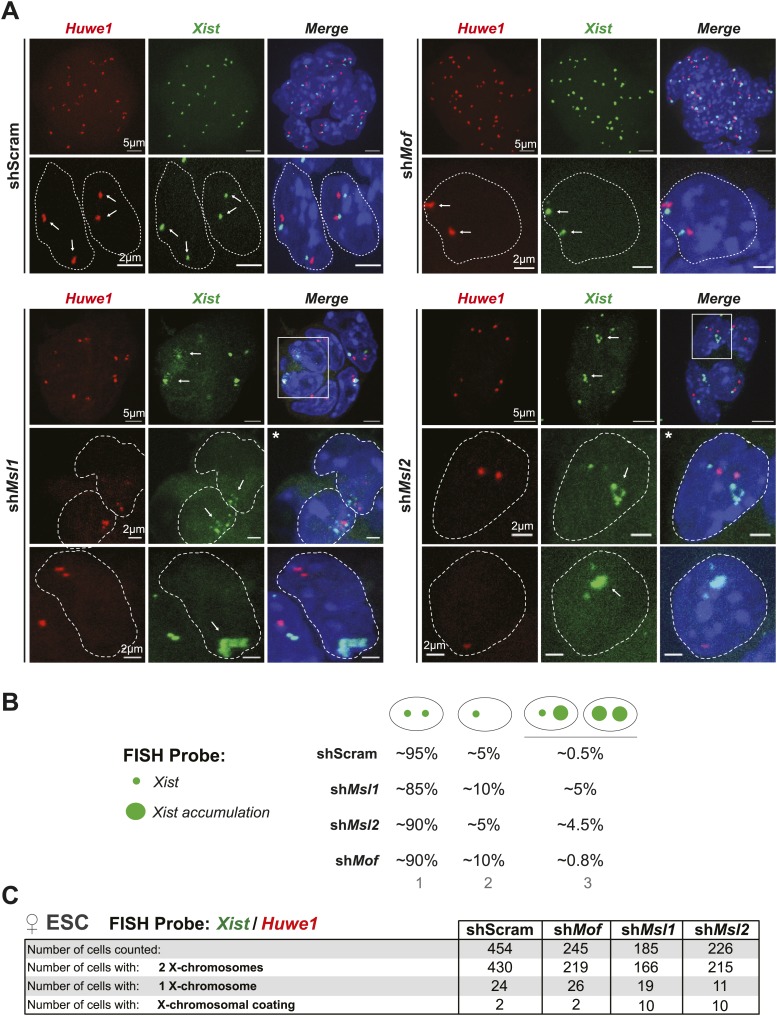
Depletion of MSL1 and MSL2 leads to occasional accumulation and spreading of *Xist* in undifferentiated ESCs. (**A**) RNA-FISH for *Huwe1* (red) and *Xist* (green) in shScrambled- (top left) and sh*Mof*- (top right), sh*Msl1*- (bottom left) and sh*Msl2*-treated (bottom right) female ESCs. Shown here are additional examples of RNA-FISH for multicellular colonies and individual cells exhibiting *Xist*-mediated coating (Figure 7A). White boxes indicate cells enlarged in Figure 7A. White arrows denote *Huwe1* and *Xist* foci. Dashed lines indicate nuclei borders. For all experiments, nuclei were counterstained with DAPI (blue). (**B**) Summary of RNA-FISH for *Xist* and *Huwe1*. The number of green dots indicates the number of X chromosomes within the cell while the larger dot indicates *Xist* accumulation. Cells were classified into three phenotypic groups with cells showing sharp, localized *Xist* signals (once or twice) or *Xist* ‘clouds’. The percentages indicate how many cells per population showed the respective phenotype. (**C**) Corresponding to Figure 7A. Summary of the total cell counts from *Xist* and *Huwe1* RNA-FISH in indicated knockdowns. **DOI:** http://dx.doi.org/10.7554/eLife.02024.023

**Figure 7—figure supplement 2.**
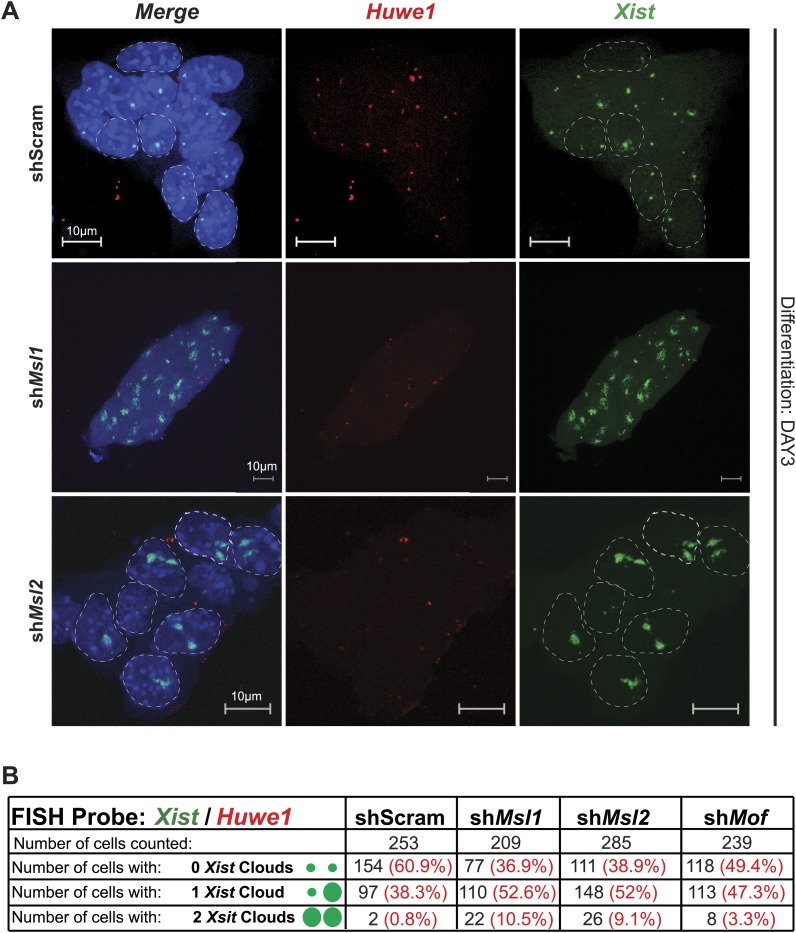
Depletion of MSL1 and MSL2 lead to enhanced *Xist* accumulation in differentiating ESCs. (**A**) RNA-FISH for *Huwe1* (red) and *Xist* (green) in shScrambled-, sh*Msl1*-, and sh*Msl2*-treated differentiating (DAY3) female ESCs. Shown here are additional examples of RNA-FISH for multicellular colonies (Figure 7C). Dashed lines indicate nuclei borders. For all experiments, nuclei were counterstained with DAPI (blue). (**B**) Corresponding to Figure 7C–E. Summary of the total cell counts from *Xist* RNA-FISH in indicated knockdowns. Percentage of cells with respective phenotype indicated in red. **DOI:** http://dx.doi.org/10.7554/eLife.02024.024

Previous studies have shown that the effects of *Tsix* depletion on *Xist* accumulation and X inactivation become fully apparent after induction of differentiation (Clerc and Avner, 1998; Debrand et al., 1999; Lee and Lu, 1999; Luikenhuis et al., 2001; Ohhata et al., 2006; Sun et al., 2006). We therefore depleted MSL1, MSL2 and MOF, and induced differentiation for 3 days by withdrawing LIF and placing the ESCs in N2B27 media. Consistent with our previous results, the induction of differentiation resulted in a stronger elevation of *Xist* RNA levels in MSL1- and MSL2-depleted cells in comparison to the scrambled control (Figure 7B). As *Tsix* expression was not affected in MOF-depleted ESCs and *Xist* levels were already high before induction of differentiation, *Xist* upregulation between day 2 and 3 of differentiation was similar to the scrambled control.

To monitor the effect on the X chromosome more closely, we next performed *Xist* RNA-FISH in MSL1-, MSL2- and MOF-depleted cells after 3 days of differentiation. All three knockdowns resulted in enhanced *Xist* accumulation and X-chromosomal coating (63.1%, 61.1% and 50.6% of all counted cells in sh*Msl1-,* sh*Msl2-,* and sh*Mof*-treated ESCs, respectively, in comparison to scrambled control with 39.1% of counted cells; see Figure 7C,D and Figure 7—figure supplement 2A,B). Interestingly, we observed that MSL1- and MSL2-depleted differentiating cells contained numerous cells with two inactive X chromosomes. The fraction of cells where both X chromosomes underwent XCI was approximately 10-fold higher in *Msl1* and *Msl2* knockdown compared to the scrambled control (Figure 7E). These results are in agreement with previously published data from homozygous *Tsix* mutants that exhibit irregular, ‘chaotic’ choice for X inactivation (Lee, 2005).

Taken together, our data establishes MSL1 and MSL2 among the key regulators of *Tsix* transcription, as the depletion of MSL proteins results in severe downregulation of *Tsix* transcription and enhanced accumulation of *Xist* during early differentiation.

## Discussion

**Figure 8.**
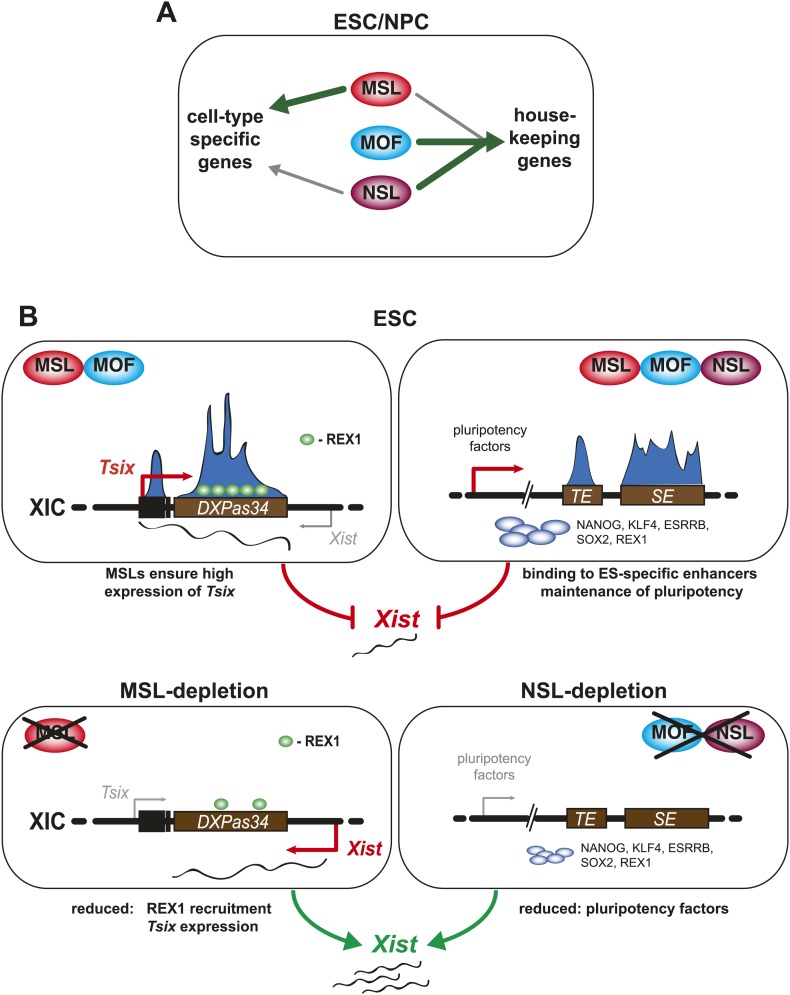
A summary model. Shared and distinct pathways by which MOF, MSLs and NSLs regulate gene expression, pluripotency, and the X inactivation center. (**A**) In this study, we have identified several modes of concurrent and independent binding of mammalian MOF, MSL and NSL proteins. We find that all complexes bind to promoters of housekeeping genes in ESCs and NPCs with NSL complex members occupying the majority of the target genes, while MOF and MSL proteins bind NSL-bound genes in a more restricted manner. Furthermore, we observe that upon differentiation, KANSL3 and MSL2 additionally occupy TSSs of different sets of cell-type-specific genes in the absence of MOF. (**B**) When we studied the functions of MSL and NSL complexes at the murine X inactivation center, we determined two basic mechanisms by which the different proteins affect the maintenance of two active X chromosomes in ESCs. (1) MSLs bind to the promoter and enhancer of *Tsix* whose transcription represses *Xist* expression. Upon depletion of MSLs, *Tsix* expression is compromised, so is REX1 recruitment to the *Tsix* locus. Consequently, *Xist* is increasingly transcribed and can occasionally accumulate. (2) In addition, MOF, MSLs, and NSLs bind to typical enhancers (TE) and super enhancers (SE) in ESCs, and notably those of pluripotency factors. In WT ESCs, the high expression of pluripotency factors is another layer of *Xist* repression. The depletions of MOF or KANSL3, but not of MSL1 or MSL2 reduce the expression of pluripotency factors involved in *Xist* repression causing a *Tsix*-independent increase of *Xist* expression. **DOI:** http://dx.doi.org/10.7554/eLife.02024.025

We present a thorough characterization of the histone acetyltransferase MOF and its two known complexes in mouse embryonic stem cells (ESCs) and neuronal progenitor cells (NPCs). We determined five basic modes of co-occurrence that revealed cell-type-specific as well as constitutive functions of the different proteins and support the notion that the NSL complex has general, housekeeping functions whereas the MSL complex predominantly performs more specialized tasks. We show that MOF and its associated proteins are involved in gene expression regulation via different means: first, they all target the promoters of housekeeping genes in a cell-type-independent manner and second, members of both complexes occupy different sets of ESC-specific enhancers that are essential for the maintenance of stem cell identity. We demonstrate the distinct and novel functions carried out by the MOF-associated complex members by revealing that both complexes contribute to the repression of X inactivation in ESCs via different means: While we establish the MSL complex as a direct regulator of *Tsix*, MOF and the NSL complex play an important role in the maintenance of pluripotency factors (Figure 8).

### Global effects of MOF are correlated with the NSL complex

Our study sheds light on the interplay between MOF and its complexes in mammals. Despite the fact that the depletion of KANSL3 does not strongly reduce global H4K16 acetylation levels, we observed strikingly similar protein and transcriptome changes in KANSL3- or MOF-depleted cells (Figure 3E–G). On the other hand, MSL1- and MSL2-depletion caused marked decreases of H4K16 acetylation (Figure 3G). This is consistent with previous reports that established MSL proteins as the main enhancers of MOF's H4K16 acetylation activity, while the NSL complex was shown to possess broader substrate specificity and can crosstalk with histone methylases (Cai et al., 2010; Kadlec et al., 2011; Zhao et al., 2013b). Unexpectedly, we observed remarkably different phenotypic changes in MSL1- or MSL2-depleted cells compared to MOF- and KANSL3-depleted cells (Figure 3E, Figure 3—figure supplement 3A). A striking example was the strong reduction of key pluripotency factors in KANSL3- and MOF-depleted cells that remain unaffected in MSL1- and MSL2-knockdowns (Figure 3G, Figure 4F). These results support the recent finding that MOF is vital for the maintenance of pluripotency (Li et al., 2012), but we furthermore show that this is an NSL- and not MSL-related function of MOF independent of H4K16 acetylation deposition.

Taken together, our data shows that while MOF is the major acetyltransferase for lysine 16 of histone 4 (Taipale et al., 2005), MSL-dependent H4K16 acetylation is one of several means through which MOF exerts its crucial biological functions. This notion was further supported by the finding that MOF predominantly binds to promoters of broadly expressed genes as part of the NSL complex and subsequently supports their transcription (Figure 3A–F). MSL1 and MSL2, on the other hand, bound to a relatively small subset of broadly expressed MOF-NSL-targeted genes that were significantly stronger expressed than those where MOF was exclusively present with NSL complex members (Figure 3B, Figure 3—figure supplement 1B). The additive effects of the complexes on gene expression were intriguing, and whether they influence each other's activity or exert their functions separately should be studied in the future. We propose that the MSL complex fine-tunes MOF's activity and ensures precise regulation of more specific targets—after all, their presence is essential for the recruitment of MOF to NSL-independent targets (Figure 5B). Our model is surprisingly similar to the picture that is emerging from *Drosophila* research where the NSL complex regulates housekeeping genes (Feller et al., 2012; Lam et al., 2012) while the MSL complex fulfills a highly specialized role on the male X chromosome (reviewed in Conrad and Akhtar, 2011).

### MSL2 and KANSL3 can contribute to transcription via enhancer binding

In addition to insights about MOF-related functions of MSL and NSL complexes, we show for the first time additional binding of MSL and NSL proteins to TSS-distal regions with enhancer characteristics. On a global scale, MOF did not yield strong enrichments for canonical enhancers; however, both MSL2 and KANSL3 showed robust signals for TSS-distal regions in ESCs, but not in NPCs, which reflected the transcriptional activity of these regions (Figure 2, Figure 4A). This apparent MOF-independent binding of the individual proteins (that tended to prefer different sets of enhancers; Figure 4B) suggests that KANSL3 and MSL2 stimulate transcription even in the absence of the histone acetyltransferase. Both proteins are in principle capable of supporting transcription: the *Drosophila* homologue of KANSL3 can directly activate transcription in vitro (Raja et al., 2010) and human MSL2 acts as an E3 ubiquitin ligase at lysine 34 of H2B (H2BK34ub) (Wu et al., 2011), which has been suggested to promote methylation of H3K4, and thus gene expression (Wu et al., 2011). Indeed, we observed several hundred genes that had been predicted to be regulated by TSS-distal binding sites of MSL2 or KANSL3 to be downregulated in the respective knockdowns with particularly high frequencies in MSL2-depleted cells (Figure 4E). It is important to note that the subset of ESC enhancers for key pluripotency factors (e.g., *Klf4*, *Sox2*) were bound concomitantly by KANSL3 and MSL2 and only the depletion of KANSL3, but not of MSL1 or MSL2 diminished protein and transcript levels of these key ESC molecules (see above). It is possible that KANSL3 could rescue loss of MSL2 at certain loci, but the exact mechanisms through which KANSL3 affects transcription via enhancer-binding need to be studied further. Furthermore, the pluripotency network and/or Mediator-related functions at super enhancers may be sufficient and dominant over MSL2 to maintain the expression of the pluripotency factors in the absence of MSL2, but may well be dependent on the function of KANSL3 at these regions.

### MSL1 and MSL2 repress X inactivation by regulating *Tsix* expression

When we specifically searched for regions where KANSL3 was not present together with MSL1 and MSL2, we found that the X inactivation center (XIC) showed numerous signals for the MSL complex (Figure 5). The XIC, a hot-spot of regulatory lncRNAs, is an X-chromosomal region that contains the main regulators of X chromosome inactivation (XCI)*.* The proper function of XIC-located non-coding RNAs is influenced by the spatial organization of the XIC and governed by a sophisticated interplay of multiple transcription factors such as pluripotency factors (Donohoe et al., 2007; Navarro et al., 2010; Deuve and Avner, 2011; Gontan et al., 2012; Nora et al., 2012).

We found that depletion of MSL1 and MSL2 severely reduced *Tsix* expression in male and in female ESCs, moderately increased *Xist* levels (Figure 6A), but left pluripotency factors unaffected (Figure 6C). In contrast, MOF-depleted cells showed downregulation of pluripotency factors and much higher *Xist* levels. Previous studies demonstrated that in undifferentiated ESCs, where pluripotency factors are highly abundant, even severe downregulation of *Tsix*, or *Tsix*-deletion has almost no effect on *Xist* transcription (Morey et al., 2001; Navarro et al., 2005; Nesterova et al., 2011). Thus, the pronounced *Xist* upregulation seen in MOF-depleted cells seems to be an indirect effect due to the downregulation of pluripotency factors, while the reduction of *Tsix* transcripts in MSL1- and MSL2-depleted cells, where pluripotency factors remain unaffected, has milder consequences on *Xist* levels.

Consequently, we could show that once ESCs are forced to initiate differentiation, the depletion of MOF has mild effects while MSL1- and MSL2-depleted cells, in which *Tsix* expression is prematurely downregulated, indeed suffer from enhanced *Xist* accumulation accompanied by ‘chaotic’ X inactivation (different numbers of inactivated X chromosomes within a population of cells; Figure 7B–E). This is consistent with the notion that the repressive potential of *Tsix* on *Xist* accumulation and the role of *Tsix* and the *DXPas34* locus in the process of counting and choice of XCI (Lee, 2005; Vigneau et al., 2006) becomes fully apparent during early stages of differentiation where additional repressive factors such as pluripotency factors are downregulated (reviewed in Rougeulle and Avner, 2004).

### Conclusion

We show that NSL and MSL complex members can function in concert to ensure proper regulation of gene expression, but our findings also strongly imply that members of both complexes have the capacity to act independently. In the case of the X inactivation center, we observe that the MOF-interacting proteins, despite engaging different regulatory means (MSL1, MSL2 through direct regulation of *Tsix*, and MOF-NSL through the pluripotency network) synergize to ensure the proper expression of the X chromosomes in undifferentiated ES cells (Figure 8). Our study sets the ground for future research to dissect the intricate interactions and specific functions of MOF and its associated major regulatory proteins in more detail.

## Materials and methods

### Cell culture

All cell culture was performed in a humidified incubator at 37°C and 5% CO_2_. The feeder-dependent mouse female embryonic stem cell line F1-21.6 was cultivated on mitomycin-C-inactivated or irradiated mouse embryonic fibroblasts (MEFs). The feeder-independent mouse male ES cell line WT26, a kind gift from the lab of Thomas Jenuwein, was cultivated on gelatin-coated dishes in ESC culture media KnockOut-DMEM (Gibco, Carlsbad, CA) supplemented with 1% L-glutamine (Gibco), 1% penicillin/streptomycin (Gibco), 1% non-essential amino acids (Gibco), 1% sodium pyruvate, 1% 2-mercaptoethanol. All ESC media contained 15% FBS and 1000 U/ml (for feeder-dependent) or 2000 U/ml (for feeder-) of leukemia inhibitory factor.

Male and female neuronal progenitor cell (NPC) lines were derived from previously mentioned ES cell lines (see below). Mouse 3T3 cells (for luciferase assays) and human HEK293-FT cells (for lentiviral production) were cultivated in DMEM (high glucose, with glutamine, Gibco) supplemented with 10% heat-inactivated serum (PAA Laboratories, North Dartmouth, MA), 1% L-glutamine, 1% penicillin/streptomycin.

### NPC differentiation

Mouse ESCs were differentiated into neuronal progenitor cells (NPC) as previously described (Conti et al., 2005; Splinter et al., 2011). In brief, 1 × 10^6^ ESCs (deprived of feeder cells) were plated on 0.1% gelatin-coated dishes in N2B27 medium and cultured for 7 days with daily media changes. The cells were then dissociated from the plate using accutase (Sigma, Germany) and 3 × 10^6^ cells were plated on a bacterial petri dish to induce formation of embryoid bodies in N2B27 medium supplemented with 10 ng/ml EGF and FGF2 (Peprotech, Rocky Hill, NJ). After 72 hr, embryoid bodies were transferred to 0.1% gelatin-coated dishes to allow adhesion and expansion of NPCs from the embryoid bodies. NPC lines were maintained in N2B27 medium supplemented with EGF and FGF2 (10 ng/ml each), on 0.1% gelatin-coated flasks. For FISH analysis, F1-21.6 ESCs were grown on gelatin-coated coverslips with a MEF-inactivated monolayer for 24 hr.

### Western blot analysis

The Invitrogen precast gel system NuPAGE was used for SDS-PAGE. The 4–12% Bi–Tris gradient gels (for proteins above 20 kDa) or 12% Bis–Tris gels (for histones and histone marks) were loaded with samples supplemented with Roti-Load 1 sample buffer. After blotting, the membranes were blocked in 5% milk with PBS + 0.3% Tween-20 (PBST) mix for at least 1 hr at room temperature. Membranes were then incubated overnight with the primary antibody in 0.5% milk with PBST at 4°C. The next day, membranes were washed three times for 10 min in PBST, incubated with a suitable HRP-coupled secondary antibody for 1 hr at room temperature, washed thrice and proteins were visualized with Lumi-Light Plus Western Blotting Substrate using the Gel Doc XR+ System.

### Immunoprecipitation assays (IP and ChIP)

For (co)immunoprecipitation (IP, co-IP) experiments, 1 ml of nuclear extract (0.5 mg/ml) was used. IPs were performed in IP buffer (25 mM HEPES pH 7.6, 150 mM KCl, 5 mM MgCl2, 0.5% Tween20, 0.2 mg/ml BSA, 1× complete protease inhibitors tablet). Extracts were incubated with 5 μg of the respective antibody or normal-rabbit/normal rat serum. For MSL1 15 μl of antibody serum was used. Extracts were incubated with the antibody for 2 hr, rotating at 4°C. Protein-A Sepharose beads (GE Healthcare, United Kingdom), blocked with 1 mg/ml yeast tRNA and 1 mg/ ml BSA (NEB, Ipswich, MA), were used for all ChIP and IP assays.

Chromatin immunoprecipitation (ChIP) assays were performed as previously described (Pauli, 2010) with minor changes. Cells were fixed in 1% molecular biology grade formaldehyde (Sigma) 9 min before being quenched with glycine (0.125 M final concentration). Cells were washed twice with ice-cold PBS and lysed on ice for 10 min with 10 ml of Farnham lysis buffer (5 mM PIPES pH 8.0, 85 mM KCl, 0.5% NP-40 + Roche Protease Inhibitor Cocktail Tablet, filtered through 0.2 micron filter unit). Lysates were transferred to a Kontes dounce tissue grinder (K885300-0015, size B) and dounced 15 times in order to break the cells and keep nuclei mostly intact. Crude nuclear prep was transferred to 15-ml falcon tube and nuclei pelleted by centrifugation at 2000 rpm at 4°C for 5 min. Nuclei were resuspended in RIPA lysis buffer (1 × PBS, 1% NP-40, 0.5% sodium deoxycholate, 0.1% SDS + Roche Protease Inhibitor Cocktail Tablet, filtered through 0.2 micron filter unit). The nuclear extract was subjected to chromatin shearing using the Diagenode Bioruptor Plus sonicator (at high setting for a total time of 25 min, 30 s ON, 30 s OFF). The sonicated mixture was centrifuged at 14,000 rpm at 4°C for 5 min and supernatant was collected. Chromatin was supplemented with 5 μg of primary antibody and incubated for 16 hr (antibodies used for ChIP are listed below). After incubation, 50 μl of 50% slurry bead solution was added for another incubation period (2 hr), then beads were washed: four times for 15 min with RIPA lysis buffer, two times for 1 min with LiCl IP wash buffer (250 mM LiCl, 10 mM Tris–HCl pH 8.0, 1 mM EDTA, 0.5% NP-40, 0.5% DOC, filtered through 0.2 micron filter unit), two times for 1 min with TE buffer (1 mM Tris–HCl pH 8.0, 1 mM EDTA, filtered through 0.2 micron filter unit). Washed beads were resuspended in 100 μl of IP elution buffer and subjected to overnight reverse cross-linking (RNase and proteinase K digestions) followed by DNA purification (DNA was purified using Minelute PCR purification kit from Qiagen, Germany). For single IP assay 50 µl of bead solution was used. Purified ChIPed DNA was subjected to qPCR amplification (Applied Biosystems, Carlsbad, CA). Input was used for normalization control. For primer pairs see Supplementary file 3.

### Antibodies

For MSL1 antibody production, a GST-mMSL1 fusion protein (C-terminal, residues 254–616) was used to immunize rabbits; the final bleed was used in experiments. Antibody specificity was verified with IP and MSL1-specific RNAi followed by Western blot analysis and ChIP assay. We used several commercial antibodies: a-KANSL1 (PAB20355; Abnova, Taiwan), a-KANSL3 (HPA035018; Sigma), a-MCRS1 (11362-1-AP; Proteintech, Chicago, IL), a-MOF (A3000992A; BETHYL Montgomery, TX), a-MSL2 (HPA003413; Sigma), a-NANOG (A300-397A; BETHYL), a-OCT3/4 (sc-5279; Santa-Cruz Dallas, TX), a-REX1 (Ab28141; Abcam, England), a-ESRRB (PP-H6705-00; Perseus Proteomics, Japan), a-KLF4 (Ab72543; Abcam), a-SOX2 (AF2018; R&D Systems, Minneapolis, MN), a-YY1 (A302-779A; BETHYL), a-RNF12/RILM (16121-1-AP; Proteintech) a-GAPDH (A300-639A; BETHYL), a-NESTIN (Ab93666; Abcam), a-CTCF (Ab70303; Abcam), a-H3 (Ab1791; Abcam), a-H4 (Ab10158; Abcam), a-H4K16ac (07-329; Millipore, Billerica, MA).

### Luciferase assays

Enhancer candidate regions (see below) were cloned into the firefly luciferase plasmids (pGL4.23; Promega, Witchburg, WI) and transfected into mouse ESCs and 3T3 fibroblasts using Lipofectamine-2000 reagent and into NPCs using LTX-PLUS reagent (Invitrogen). Transfections were performed according to the manufacturer's guidelines except for using a 1:6 DNA to Lipofectamine ratio. Cells were seeded 1 day prior to transfection to achieve 70–80% confluency at the time of transfection. Next, cells were fed with antibiotics-free medium (ES medium with LIF for ESCs and OPTIMEM for NPCs and 3T3s) at least 30 min before transfection and the medium was changed back 6–8 hr after transfection (basal neural medium with FGF and EGF for NPCs). 100 ng of firefly construct with the cloned candidate region was co-transfected with 1 ng of renilla luciferase construct (pRL-TK of Promega) per 96-well and harvested for luciferase assay after 24 hr. Cells were harvested for luciferase assay 24 hr after transfection. The Dual Luciferase Kit (Promega) was used according to the manufacturer's protocol but with reduced substrate volumes of LARII and Stop&Glo reagents (50 µl per well of a 96-well plate with 10 µl cell lysate). Luminescence was measured by using Mithras plate reader (Berthold, Germany).

The transfection efficiency was normalized by firefly counts divided by the renilla counts. The fold enhancement value was calculated by an additional normalization to minimal promoter alone activities in each experiment (the graphs represent at least three independent experiments that were performed in technical triplicates each with error bars representing standard error of the mean). The following enhancer candidate regions were amplified from mouse genomic DNA by PCR and cloned into BamHI-SalI sites (downstream of luciferase gene) of firefly luciferase plasmid pGL4.23 (Promega):

Intron of *Esrrb* (chr12:87,842,537-87,843,719) with primers introducing BamHI and XhoI sites: ATAGGATCCGAAGTAATTGTCTATTGTATCAG (forward), TATCTCGAGAAGAAGAAAGACTGTGTTCAACTCC (reverse).

Upstream of *Lefty* (chr1: 182854617-182855516) with primers introducing BamHI and SalI sites: ATAGGATCCCTTGCGGGGGATATGAGGC (forward), TATGTCGACCTGGGCCTTTCTAAGGC (reverse).

Upstream of *Trim28* (*Kap1*) (chr18: 34309039-34310140) with primer introducing BamHI and SalI sites: ATAGGATCCGAGGACTATTTGAAGGATCTATT (forward), TATGTCGACCTCACTCCCCAACCTCCATTTC (reverse).

Upstream of *Apc* (chr18: 34309039-34310140) with primers introducing BamHI and SalI sites: ATAGGATCCCTGAGCAATGCTCTTCCTCACAAGC (forward), TATGTCGACTTATACTCCAAATAGAATTGTCTG (reverse).

Intron of *Tbx3* (chr5: 120129690- 120130617) with primers introducing BamHI and SalI sites: ATAGGATCCATAAATAAATAAATAAATATCTGATTG (forward), TATGTCGACCGCGAGTCTGGCGATGCCTTGTC (reverse).

### RNA extraction followed by cDNA synthesis and quantitative real time PCR

cDNA was synthesized from 500 ng–1 µg of total RNA (extracted from circa 1 million cells using Rneasy kit, Qiagen) with random hexamers using SuperScript-III First Strand Synthesis kit (Invitrogen). The qPCRs were carried in a total reaction volume of 25 µl containing 0.5–1 µl of cDNA, 0.4 µmol of forward and reverse primer mix and 50% 2 × SYBR Green PCR Master Mix (Roche). Gene expression was normalized to multiple controls (*RplP0* or *Hprt*), using the 7500 software V2.0.4 for analysis (Applied Biosystems). For primer pairs used for expression profiling see Supplementary file 3C.

### Lentiviral-based RNAi in ESCs

shRNA constructs were either obtained from Sigma in pLKO.1 or designed using Genscript and cloned (please see below for details). For cloning, forward and reverse complimentary DNA oligonucleotides (Eurofins MWG Operon, Germany) designed to produce AgeI (5′) and EcoRI (3′) overhangs were annealed at a final concentration of 2 µM in NEBuffer. The pLKO.1-puro plasmid was digested with AgeI and EcoRI, ligated to the annealed oligonucleotides, and transformed into HB101 competent cells (Promega). Plasmid DNA was purified using the QIAprep Spin Miniprep kit (Qiagen), and the sequence was validated.

For production of lentiviral particles, 70% confluent HEK293FT cells in a 10-cm tissue culture plate were co-transfected with 3.33 µg lentiviral construct, 2.5 µg psPAX2 packaging plasmid and 1 µg pMD2.G envelope plasmid using Lipofectamine-2000 reagent (Invitrogen). To transduce ESCs, either concentrated or diluted lentiviral particles were used. For concentrated lentivirus, transfections were scaled up and OPTIMEM (Invitrogen) added to the HEK293FT cells following transfection and the lentiviral supernatant collected at 48 and 72 hr post-infection. This was then concentrated using Amicon Ultra-15 centrifugal filter units (Millipore) and added to ESC media supplemented with LIF and 10 µg/ml polybrene (Millipore). For diluted lentivirus, ESC media without LIF was added to the HEK293FT cells and the lentiviral supernatant was collected after 48 hr, filtered through 0.22 µm filters (Whatmann), and added 1:1 with fresh ESC media supplemented with LIF and polybrene to the ESCs. ESCs were then subjected to selection with 1.0 µg/ml puromycin, passaged once, and harvested on day 3, 4, 5 or 6 of knockdown depending on the experiment (the numbers of days are indicated in the corresponding results section).

The following shRNA sequences were used for the knockdowns:

CCGGCCTAAGCACTCTCCCATTAAACTCGAGTTTAATGGGAGAGTGCTTAGGTTTTTG (sh*Msl1*, SIGMA, TRCN0000241378),
CCGGCCCAGTCTCTTAGCCATAATGCTCGAGCATTATGGCTAAGAGACTGGGTTTTTG (sh*Msl2*, SIGMA, TRCN0000243429),
CCGGAAGGCCGAGAAGAATTCTATCTCGAGATAGAATTCTTCTCGGCCTTTTTTTG (sh*Mof*, GENSCRIPT designed),
CCGGCTCCAGTCCTCTTCGTCATTGCTCGAGCAATGACGAAGAGGACTGGAGTTTTTG (sh*Kansl3*, SIGMA, TRCN0000266995),
CCGGAAGTGGCGCCTTAGCAACAACCTCGAGGTTGTTGCTAAGGCGCACTTTTTTTG (sh*Mcrs1*, GENSCRIPT designed),
CCGGCAACAAGATGAAGAGCACCAACTCGAGACAATTCGGAAGAAATCTGAGCTTTTTG (Non-targeting control, SIGMA, SHC002).

### Cell proliferation assay

Cells treated with respective shRNAs and scramble control were performed as described earlier in feeder-free W26 mouse ESCs. The cell count was monitored for 6 days post knockdown at 24-hr intervals. In brief, after 4 days of knockdown six sets of 0.4 × 10^4^ cells per well were seeded in triplicates in a 12-well gelatinized plate. The cells were grown in ES cell culture medium supplemented with 2000 U/ml LIF and 1 μg/ml puromycin; the medium was changed every 24 hr. For counting, cells were trypsinized and counted using the Neubauer hemocytometer.

### Alkaline phosphatase staining

Detection of alkaline phosphatase, a surface marker and indicator of undifferentiated ESCs, was performed using the following method: feeder-free W26 ESCs were transduced (4 days) with scramble or the shRNAs against the genes of interest. Cells were washed twice with PBS followed by fixation with 4% PFA for 2–3 min. The cells were washed twice with PBS and stained for 20 min with staining solution (25 mM Tris-Maleic acid buffer pH 9.0, 0.4 mg/ml α-Naphthyl Phosphate (Sigma), 1 mg/ml Fast Red TR Salt (Sigma), 8 mM MgCl_2_, 0.01% Deoxycholate, 0.02% NP40). The reaction was stopped by washing with water followed by two washes with 1 × PBS.

### RNA extraction for RNA-seq

Total RNA was extracted from WT26 ESCs and NPCs as biological triplicates using TRIzol Reagent and treated with the TURBO Dnase kit (Ambion).

For RNA-seq of knockdowns, feeder-free WT26 ESCs were transduced with shRNAs specific for *Msl1*, *Msl2*, *Mof*, *Kansl3* and control shRNA as biological triplicates as described above. Briefly, following transduction for 24 hr, cells were washed with PBS thrice to remove the viral supernatant and subjected to puromycin selection (1.5 µg/ml) for 24 hr. In the case of *Msl1/2*, *Mof*, control shRNA the cells were maintained in puromycin selection for 4 days and in case of *Kansl3,* the cells were maintained in puromycin-selection for 84 hr. An additional set of control shRNA was performed alongside with *Kansl3* for 84 hr. Total RNA from all the shRNA-treated cells was extracted using TRIzol Reagent and the samples were treated with DNase using the TURBO DNase kit (Ambion). The quality of the RNA was analyzed using the Bioanalyzer and samples with RIN values between 9 and 10 were used for RNA-seq. For RNA-seq analysis, cDNA libraries were prepared using the Illumina TruSeq Stranded mRNA kit with 3 μg DNase-treated samples. 

### RNA-FISH

*Xist* and *Huwe1* probes were described previously (Chow et al., 2010). *Tsix* was detected with a *DXPas34* plasmid (Debrand et al., 1999). Approximately 1 × 10^5^ of F1-21.6 ESCs were plated on gelatin-coated coverslips and incubated for 24 to 48 hr. After fixation and permeabilization, coverslips with cells were washed and stored in 70% EtOH at −20°C. Then the coverslips were dehydrated in 80%, 95%, and 100% EtOH (5 min each) and briefly air-dried. FISH probes were labeled by nick translation (Abbott) with Spectrum Red-dUTP or Spectrum Green-dUTP following the manufacturer's instructions. Labeled probes were precipitated in the presence of salmon sperm (10 μg) and Cot-1 DNA (3 μg), denatured and competed with Cot-1 DNA for 45 min at 37°C. Cells were then directly hybridized with labeled probes at 37°C overnight. Next, coverslips were washed three times in 50% formamide/2 × SSC followed by three washes in 2 × SSC at 42°C. Cells were stained with DAPI (0.2 mg/ml).

### Immunofluorescence staining (against NESTIN)

Approximately 1 × 10^5^ of male W26 ESCs and NPCs were plated on gelatin-coated coverslips and incubated for 24 hr. The cells were washed twice with PBS and fixed with pre-warmed 4% formaldehyde for 8 min at 37°C. Next, cells were washed thrice with PBS, 5 min each at room temperature and incubated in Permeabilization buffer (1 × PBS, 0.2% Triton X-100) for 5 min at room temperature. After permeabilization cells were incubated in Blocking buffer (1 × PBS, 5% BSA, 0.05% Triton-X100) for 30 min, stained for 1 hr with primary antibody (rabbit polyclonal a-NESTIN, 1:500). Next, cells were washed thrice with Wash buffer (1 × PBS, 0.05% Triton-X100) and incubated in 10% goat normal serum solution (Invitrogen) for 20 min. Secondary antibody (goat anti-rabbit Alexa Fluor-488, 1:1000) was added on coverslips and incubated for 45 min.

### Microscopy

We used a spinning disk confocal microscope (Observer 1/Zeiss) with Plan Apochromat 63x1.4-oil objective for magnification. 500 ms exposure time was used for all lasers. Sequential z-axis images were collected in 0.5 μm steps. ZEN Blue software was used for image analysis.

### Sequencing

All samples were sequenced by the Deep Sequencing Unit (MPI-IE, Freiburg) using Illumina HiSeq2000. Library preparation was carried out following Illumina standard protocols for paired-end sequencing (50 bp reads). All raw reads can be found in the GEO database under the accession number GSE51746.

### RNA-seq data processing

RNA-seq reads were mapped to Ensembl annotation NCBIM37/mm9 using TopHat2 (Kim et al., 2013) with the options mate-inner-dist, mate-std-dev and library-type (fr-firststrand). The distance between read mates (mate-inner-dist and mate-std-dev) were assessed individually for each sequenced library based on the output of the sequencer for average fragment size and CV value.

For FPKM value generation, cufflinks (version 2.1.1) was used for each transcript in each condition (three replicates for ESC and NPC) with default parameters; CummeRbund was used for quality checks and data access (Trapnell et al., 2013). Based on the distribution of FPKM values, active genes were defined as transcripts with mean FPKM ≥4 (average over the replicates).

### Differential gene expression analysis

After mapping of the RNA-seq reads from the shRNA-treated samples (including scrambled control), the reads that mapped to the genome were counted using htseq-count (doi: 10.1101/002824) with the stranded option set to reverse. The annotations present in the *Mus musculus* gtf file from the ENSEMBL release 67 were used as reference for counting.

DESeq2 was used for differential expression analysis (Anders and Huber, 2010). In this analysis, all libraries from knockdown cells were compared in a pairwise manner with its corresponding scrambled shRNA samples. Within the DESeq2 workflow, the cooks–cutoff parameter was set to ‘FALSE’ and the genes with an adjusted p-value ≤0.01 were defined as significantly affected.

### ChIP-seq analysis

#### Read mapping and normalizations

After mapping of the paired-end reads to the mouse genome (mm9) using bowtie version 2 (Langmead and Salzberg, 2012), we filtered for duplicate reads, reads with mapping qualities smaller than 2 and ambiguously mapped reads using samtools (Li et al., 2009). We also removed reads mapping to the mitochondrial genome and ‘random’ chromosomes, as well as known major satellites and duplicated genome regions to avoid coverage biases.

For normalization procedures, several modules of the deepTools suite (https://deeptools.github.io) were used (Ramirez et al., 2014). To ensure a fair comparison between all data sets, first, the GC bias of all mapped reads was determined and, if necessary, corrected so that input and ChIP samples had similar GC distributions of their reads (correctGCbias module). In addition, all aligned read files were corrected for sequencing depth using the signal extraction method proposed by Diaz et al. (2012) and normalized to the cell-type-specific input (bamCompare module).

### Peak calling and replicate handling

MACS (version 1.4) was used for peak calling on every sample individually and on the merged files of two replicates (Zhang et al., 2008). Only peaks present in both replicates were considered, using the borders and summits defined by peak calling results for the merged replicates. In addition, peaks with −10log_10_(p-values) lower than 50 and false-discovery rate values greater than 0.1% were excluded from down-stream analyses.

### Annotation used for genome-wide analyses

We used the RefSeq gene list for genome version mm9/NCBI37. Unless specified otherwise, alternative transcription start sites were scored as individual TSS in the respective analyses. The list of genes with homologues in different species was downloaded from HomoloGene and subsequently filtered for pairs of mouse and fly genes that belong to the same clusters of homology ID. CpG island information was downloaded from the UCSC Genome Browser (Wu et al., 2010), mean observed over expected CpG ratios were extracted for the TSSs ± 0.5 kb using UCSC tools.

### Clustering

For Figure 2, a matrix containing the normalized ChIP-seq signals for all peaks was generated as follows: first, the union of peaks was created using mergeBed from the bedtools suite (Quinlan and Hall, 2010); then each region was binned to 2 kb and the normalized ChIP values were extracted in 50 bp windows. The ChIP signal values were rank-transformed, converted into euclidean distances using the R function ‘dist’ and subsequently ordered according to their similarity by the ‘hclust’ function (using Ward's method). The resulting dendrogram was pruned to 2 to 10 clusters for which the individual ChIP signals for unscaled regions were extracted (Figure 2). Visual inspection revealed no striking differences of the binding patterns between the individual clusters for more than 5 clusters.

The 3 clusters displayed in the lower part of Figure 4—figure supplement 2B were obtained similarly: first, a matrix was generated that contained the normalized ChIP-seq values of MOF, p300, H3K4me1 and DNase hypersensitivity sites for all regions of cluster D that did not overlap with ESC enhancers. The regions were then scaled to 1400 bp and mean values were computed for 50 bp bins using the computeMatrix module of deepTools (Ramirez et al., 2014). Further processing was done as described above; the resulting dendrogram was pruned to *k* = 3 and the enrichments of the different factors were computed and visualized for 10 kb regions using the heatmapper module of deepTools.

### GO term analysis

For GO term analyses, we used two approaches: the web interface of DAVID (Huang da et al., 2009) and GREAT (McLean et al., 2010).

For DAVID, we determined genes overlapping with the peaks of the individual ChIP-seq samples (TSS region ± 500 bp) and supplied the corresponding RefSeq-IDs. The background list contained the union of all TSSs bound by at least one ChIPed protein. We used the Functional Annotation Clustering tool, filtered with the option ‘high stringency’ and manually grouped the returned clusters of gene functions with enrichment scores above 1.3 into even broader terms.

To assess the GO terms of genes that might be regulated by the TSS-distal binding sites of MOF, MSL1, MSL2, KANSL3 and MCRS1, we used GREAT (McLean et al., 2010) with the mouse genome as the background data set and default settings. We obtained the top-ranked biological processes of the genes suggested to be *cis*-regulated by the regions combined in cluster D (Figure 2).

### Analysis of transcription factor binding sites

For the analysis of enriched transcription factor binding sites, we used the R package ChIPEnrich (http://sartorlab.ccmb.med.umich.edu/chip-enrich) and TRAP (Thomas-Chollier et al., 2011). The ChIPEnrich package takes peak regions as input and uses a logistic regression approach to test for gene set enrichments while normalizing for mappability and locus length. We supplied the regions belonging to the individual clusters of binding (A–E from Figure 2) and obtained the corresponding enriched transcription factors.

To plot the occurrences of the SMAD3 motif (V$SMAD3_Q6, TRANSFAC name M00701; Figure 4—figure supplement 3C), TRAP was used with the following command to generate a bedgraph file where the log likelihood of a SMAD3 motif occurrence is stored for the entire genome: ANNOTATEv3.04_source/Release/ANNOTATE_v3.04 -s mm9.fa --pssm /transfac.pssm -g 0.5 --ttype balanced -name M00701 -d | awk 'BEGIN{OFS=t}{print $1, $4+7, $4+8, $6}' > SMAD3.pssm.bedgraph.

### Heatmap visualizations and summary plots

Heatmaps displaying normalized read densities of ChIP-seq samples, % methylated CpGs and SMAD3 motif score (Figures 2, 3C, 4A, Figure 4—figure supplement 2B and 3) were generated with the computeMatrix and heatmapper modules of the deepTools package (Ramirez et al., 2014) with ‘reference-point’ mode. Heatmaps of fractions of overlapped regions as in Figure 3—figure supplement 1 and Figure 4B as well as log_2_ fold changes (knockdown/control) from RNA-seq experiments (Figure 3E) were generated with the function ‘heatmap.2’ from the R gplots package.

The values underlying the summary plots such as the meta-gene and meta-enhancer plots in Figures 3D and 4B, Figure 3—figure supplement 2B, Figure 4—figure supplement 1B, 2A,C were generated with the computeMatrix module of the deepTools package using either ‘reference-point’ or ‘scale-regions’ mode and were visualized with the R package ggplots2.

### Working with genomic intervals

For general assessments of overlaps between bed-files and to extract scores for defined regions the bedTools suite (Quinlan and Hall, 2010) and UCSC tools (Kuhn et al., 2013) were used. The snapshots of the binding profiles were obtained with IGV browser (Thorvaldsdottir et al., 2013).

### Target definitions

For each knockdown condition for which RNA-seq data had been generated (see above), significantly affected genes were used (adjusted p-value ≤0.01, see above for differential gene expression analysis). Then they were subdivided into TSS- (ChIP-seq peak overlap with TSS ±1 kb), TSS-distal- (ChIP-seq peaks not overlapping with TSS ±1 kb) and non-targets (neither TSS overlap nor part of TSS-distal list). A gene was classified as TSS-distally regulated when at least one of the following criteria was true:

1. TSS-distal peaks overlapped its published super or typical enhancer (Whyte et al., 2013)
2. TSS-distal peaks were predicted by GREAT (McLean et al., 2010) to regulate the respective gene
3. TSS-distal peaks overlapped with at least one intron

Genes were defined as MSL targets when peaks of MOF and MSL1|MSL2 were overlapping at the TSS ±1 kb or TSS-distal peaks were predicted to regulate the same putative target gene. NSL targets were defined the same way, but with co-occurrences of peaks from MOF and KANSL3|MCRS1.

## Additional files

Supplementary file 1. MOF/MSL/NSL ChIP-seq statistics. **DOI:** http://dx.doi.org/10.7554/eLife.02024.026

Supplementary file 2. Detailed information about publicly available genome-wide resources used in this study. **DOI:** http://dx.doi.org/10.7554/eLife.02024.027

Supplementary file 3. Lists of primer pairs used in ChIP-qPCR and RT-PCR analyses. **DOI:** http://dx.doi.org/10.7554/eLife.02024.028

### Major dataset

The following dataset was generated:

DuendarF, 2013, MOF-associated complexes ensure stem cell identity and Xist repression, http://www.ncbi.nlm.nih.gov/geo/query/acc.cgi?acc=GSE57701, Publicly available at NCBI GEO.

## References

[bib1] Akhtar A Becker PB 2000 Activation of transcription through histone H4 acetylation by MOF, an acetyltransferase essential for dosage compensation in Drosophila. Molecular Cell 5:367–375. doi: 10.1016/S1097-2765(00)80431-1 10882077

[bib2] Anders S Huber W 2010 Differential expression analysis for sequence count data. Genome Biology 11:R106. doi: 10.1186/gb-2010-11-10-r106 20979621 PMC3218662

[bib3] Ang YS Tsai SY Lee DF Monk J Su J Ratnakumar K Ding J Ge Y Darr H Chang B Wang J Rendl M Bernstein E Schaniel C Lemischka IR 2011 Wdr5 mediates self-renewal and reprogramming via the embryonic stem cell core transcriptional network. Cell 145:183–197. doi: 10.1016/j.cell.2011.03.003 21477851 PMC3097468

[bib4] Anguera MC Ma W Clift D Namekawa S Kelleher RJ III Lee JT 2011 Tsx produces a long noncoding RNA and has general functions in the germline, stem cells, and brain. PLOS Genetics 7:e1002248. doi: 10.1371/journal.pgen.1002248 21912526 PMC3164691

[bib5] Cai Y Jin J Swanson SK Cole MD Choi SH Florens L Washburn MP Conaway JW Conaway RC 2010 Subunit composition and substrate specificity of a MOF-containing histone acetyltransferase distinct from the male-specific lethal (MSL) complex. The Journal of Biological Chemistry 285:4268–4272. doi: 10.1074/jbc.C109.087981 20018852 PMC2836030

[bib6] Chow JC Ciaudo C Fazzari MJ Mise N Servant N Glass JL Attreed M Avner P Wutz A Barillot E Greally JM Voinnet O Heard E 2010 LINE-1 activity in facultative heterochromatin formation during X chromosome inactivation. Cell 141:956–969. doi: 10.1016/j.cell.2010.04.042 20550932

[bib7] Chureau C Chantalat S Romito A Galvani A Duret L Avner P Rougeulle C 2011 Ftx is a non-coding RNA which affects Xist expression and chromatin structure within the X-inactivation center region. Human Molecular Genetics 20:705–718. doi: 10.1093/hmg/ddq516 21118898

[bib8] Clerc P Avner P 1998 Role of the region 3' to Xist exon 6 in the counting process of X-chromosome inactivation. Nature Genetics 19:249–253. doi: 10.1038/924 9662396

[bib9] Cohen DE Davidow LS Erwin JA Xu N Warshawsky D Lee JT 2007 The DXPas34 repeat regulates random and imprinted X inactivation. Developmental Cell 12:57–71. doi: 10.1016/j.devcel.2006.11.014 17199041

[bib10] Conrad T Akhtar A 2011 Dosage compensation in Drosophila melanogaster: epigenetic fine-tuning of chromosome-wide transcription. Nature Reviews Genetics 13:123–134. doi: 10.1038/nrg3124 22251873

[bib11] Conti L Pollard SM Gorba T Reitano E Toselli M Biella G Sun Y Sanzone S Ying QL Cattaneo E Smith A 2005 Niche-independent symmetrical self-renewal of a mammalian tissue stem cell. PLOS Biology 3:e283. doi: 10.1371/journal.pbio.0030283 16086633 PMC1184591

[bib12] Creyghton MP Cheng AW Welstead GG Kooistra T Carey BW Steine EJ Hanna J Lodato MA Frampton GM Sharp PA Boyer LA Young RA Jaenisch R 2010 Histone H3K27ac separates active from poised enhancers and predicts developmental state. Proceedings of the National Academy of Sciences of the United States of America 107:21931–21936. doi: 10.1073/pnas.1016071107 21106759 PMC3003124

[bib13] Cui G Park S Badeaux AI Kim D Lee J Thompson JR Yan F Kaneko S Yuan Z Botuyan MV Bedford MT Cheng JQ Mer G 2012 PHF20 is an effector protein of p53 double lysine methylation that stabilizes and activates p53. Nature Structural & Molecular Biology 19:916–924. doi: 10.1038/nsmb.2353 PMC3454513 22864287

[bib14] Debrand E Chureau C Arnaud D Avner P Heard E 1999 Functional analysis of the DXPas34 locus, a 3' regulator of Xist expression. Molecular and Cellular Biology 19:8513–8525 10567576 10.1128/mcb.19.12.8513 PMC84967

[bib16] Deuve JL Avner P 2011 The coupling of X-chromosome inactivation to pluripotency. Annual Review of Cell and Developmental Biology 27:611–629. doi: 10.1146/annurev-cellbio-092910-154020 21801017

[bib17] Diaz A Park K Lim DA Song JS 2012 Normalization, bias correction, and peak calling for ChIP-seq. Statistical Applications in Genetics and Molecular Biology 11 Article 9. doi: 10.1515/1544-6115.1750 PMC3342857 22499706

[bib18] Donohoe ME Silva SS Pinter SF Xu N Lee JT 2009 The pluripotency factor Oct4 interacts with Ctcf and also controls X-chromosome pairing and counting. Nature 460:128–132. doi: 10.1038/nature08098 19536159 PMC3057664

[bib19] Donohoe ME Zhang LF Xu N Shi Y Lee JT 2007 Identification of a Ctcf cofactor, Yy1, for the X chromosome binary switch. Molecular Cell 25:43–56. doi: 10.1016/j.molcel.2006.11.017 17218270

[bib20] Farre D Bellora N Mularoni L Messeguer X Alba MM 2007 Housekeeping genes tend to show reduced upstream sequence conservation. Genome Biology 8:R140. doi: 10.1186/gb-2007-8-7-r140 17626644 PMC2323216

[bib21] Feller C Prestel M Hartmann H Straub T Soding J Becker PB 2012 The MOF-containing NSL complex associates globally with housekeeping genes, but activates only a defined subset. Nucleic Acids Research 40:1509–1522. doi: 10.1093/nar/gkr869 22039099 PMC3287193

[bib22] Gendrel AV Attia M Chen CJ Diabangouaya P Servant N Barillot E Heard E 2014 Developmental dynamics and disease potential of random monoallelic gene expression. Developmental Cell 28:366–380. doi: 10.1016/j.devcel.2014.01.016 24576422

[bib23] Gontan C Achame EM Demmers J Barakat TS Rentmeester E Van IW Grootegoed JA Gribnau J 2012 RNF12 initiates X-chromosome inactivation by targeting REX1 for degradation. Nature 485:386–390. doi: 10.1038/nature11070 22596162

[bib24] Gupta A Guerin-Peyrou TG Sharma GG Park C Agarwal M Ganju RK Pandita S Choi K Sukumar S Pandita RK Ludwig T Pandita TK 2008 The mammalian ortholog of Drosophila MOF that acetylates histone H4 lysine 16 is essential for embryogenesis and oncogenesis. Molecular and Cellular Biology 28:397–409. doi: 10.1128/MCB.01045-07 17967868 PMC2223300

[bib25] Gupta A Hunt CR Pandita RK Pae J Komal K Singh M Shay JW Kumar R Ariizumi K Horikoshi N Hittelman WN Guha C Ludwig T Pandita TK 2013 T-cell-specific deletion of Mof blocks their differentiation and results in genomic instability in mice. Mutagenesis 28:263–270. doi: 10.1093/mutage/ges080 23386701 PMC3630520

[bib26] Hallacli E Lipp M Georgiev P Spielman C Cusack S Akhtar A Kadlec J 2012 Msl1-mediated dimerization of the dosage compensation complex is essential for male X-chromosome regulation in Drosophila. Molecular Cell 48:587–600. doi: 10.1016/j.molcel.2012.09.014 23084835

[bib27] Hu G Kim J Xu Q Leng Y Orkin SH Elledge SJ 2009 A genome-wide RNAi screen identifies a new transcriptional module required for self-renewal. Genes & Development 23:837–848. doi: 10.1101/gad.1769609 19339689 PMC2666338

[bib28] Huang da W Sherman BT Lempicki RA 2009 Systematic and integrative analysis of large gene lists using DAVID bioinformatics resources. Nature Protocols 4:44–57. doi: 10.1038/nprot.2008.211 19131956

[bib29] Jeon Y Lee JT 2011 YY1 tethers Xist RNA to the inactive X nucleation center. Cell 146:119–133. doi: 10.1016/j.cell.2011.06.026 21729784 PMC3150513

[bib30] Jonkers I Barakat TS Achame EM Monkhorst K Kenter A Rentmeester E Grosveld F Grootegoed JA Gribnau J 2009 RNF12 is an X-Encoded dose-dependent activator of X chromosome inactivation. Cell 139:999–1011. doi: 10.1016/j.cell.2009.10.034 19945382

[bib31] Kadlec J Hallacli E Lipp M Holz H Sanchez-Weatherby J Cusack S Akhtar A 2011 Structural basis for MOF and MSL3 recruitment into the dosage compensation complex by MSL1. Nature Structural & Molecular Biology 18:142–149. doi: 10.1038/nsmb.1960 21217699

[bib32] Kim D Pertea G Trapnell C Pimentel H Kelley R Salzberg SL 2013 TopHat2: accurate alignment of transcriptomes in the presence of insertions, deletions and gene fusions. Genome Biology 14:R36. doi: 10.1186/gb-2013-14-4-r36 23618408 PMC4053844

[bib33] Koolen DA Kramer JM Neveling K Nillesen WM Moore-Barton HL Elmslie FV Toutain A Amiel J Malan V Tsai AC Cheung SW Gilissen C Verwiel ET Martens S Feuth T Bongers EM De Vries P Scheffer H Vissers LE De Brouwer AP Brunner HG Veltman JA Schenck A Yntema HG De Vries BB 2012 Mutations in the chromatin modifier gene KANSL1 cause the 17q21.31 microdeletion syndrome. Nature Genetics 44:639–641. doi: 10.1038/ng.2262 22544363

[bib34] Kruse JP Gu W 2009 MSL2 promotes Mdm2-independent cytoplasmic localization of p53. The Journal of Biological Chemistry 284:3250–3263. doi: 10.1074/jbc.M805658200 19033443 PMC2631942

[bib35] Kuhn RM Haussler D Kent WJ 2013 The UCSC genome browser and associated tools. Briefings in Bioinformatics 14:144–161. doi: 10.1093/bib/bbs038 22908213 PMC3603215

[bib36] Kumar R Hunt CR Gupta A Nannepaga S Pandita RK Shay JW Bachoo R Ludwig T Burns DK Pandita TK 2011 Purkinje cell-specific males absent on the first (mMof) gene deletion results in an ataxia-telangiectasia-like neurological phenotype and backward walking in mice. Proceedings of the National Academy of Sciences of the United States of America 108:3636–3641. doi: 10.1073/pnas.1016524108 21321203 PMC3048124

[bib37] Lam KC Mühlpfordt F Vaquerizas JM Raja SJ Holz H Luscombe NM Manke T Akhtar A 2012 The NSL complex regulates housekeeping genes in Drosophila. PLOS Genetics 8:e1002736. doi: 10.1371/journal.pgen.1002736 22723752 PMC3375229

[bib38] Landolin JM Johnson DS Trinklein ND Aldred SF Medina C Shulha H Weng Z Myers RM 2010 Sequence features that drive human promoter function and tissue specificity. Genome Research 20:890–898. doi: 10.1101/gr.100370.109 20501695 PMC2892090

[bib39] Langmead B Salzberg SL 2012 Fast gapped-read alignment with Bowtie 2. Nature Methods 9:357–359. doi: 10.1038/nmeth.1923 22388286 PMC3322381

[bib40] Lee JT 2005 Regulation of X-chromosome counting by Tsix and Xite sequences. Science 309:768–771. doi: 10.1126/science.1113673 16051795

[bib41] Lee JT Lu N 1999 Targeted mutagenesis of Tsix leads to nonrandom X inactivation. Cell 99:47–57. doi: 10.1016/S0092-8674(00)80061-6 10520993

[bib42] Lee KK Workman JL 2007 Histone acetyltransferase complexes: one size doesn't fit all. Nature Reviews Molecular Cell Biology 8:284–295. doi: 10.1038/nrm2145 17380162

[bib43] Li H Handsaker B Wysoker A Fennell T Ruan J Homer N Marth G Abecasis G Durbin R, Genome Project Data Processing Subgroup 2009 The sequence Alignment/Map format and SAMtools. Bioinformatics 25:2078–2079. doi: 10.1093/bioinformatics/btp352 19505943 PMC2723002

[bib44] Li X Li L Pandey R Byun JS Gardner K Qin Z Dou Y 2012 The histone acetyltransferase MOF is a key regulator of the embryonic stem cell core transcriptional network. Cell Stem Cell 11:163–178. doi: 10.1016/j.stem.2012.04.023 22862943 PMC3413170

[bib45] Luikenhuis S Wutz A Jaenisch R 2001 Antisense transcription through the Xist locus mediates Tsix function in embryonic stem cells. Molecular and Cellular Biology 21:8512–8520. doi: 10.1128/MCB.21.24.8512-8520.2001 11713286 PMC100014

[bib46] McLean CY Bristor D Hiller M Clarke SL Schaar BT Lowe CB Wenger AM Bejerano G 2010 GREAT improves functional interpretation of cis-regulatory regions. Nature Biotechnology 28:495–501. doi: 10.1038/nbt.1630 PMC4840234 20436461

[bib47] Mendjan S Taipale M Kind J Holz H Gebhardt P Schelder M Vermeulen M Buscaino A Duncan K Mueller J Wilm M Stunnenberg HG Saumweber H Akhtar A 2006 Nuclear pore components are involved in the transcriptional regulation of dosage compensation in Drosophila. Molecular Cell 21:811–823. doi: 10.1016/j.molcel.2006.02.007 16543150

[bib48] Morey C Arnaud D Avner P Clerc P 2001 Tsix-mediated repression of Xist accumulation is not sufficient for normal random X inactivation. Human Molecular Genetics 10:1403–1411. doi: 10.1093/hmg/10.13.1403 11440993

[bib49] Nagy Z Riss A Fujiyama S Krebs A Orpinell M Jansen P Cohen A Stunnenberg HG Kato S Tora L 2010 The metazoan ATAC and SAGA coactivator HAT complexes regulate different sets of inducible target genes. Cellular and Molecular Life Sciences 67:611–628. doi: 10.1007/s00018-009-0199-8 19936620 PMC11115597

[bib50] Navarro P Chambers I Karwacki-Neisius V Chureau C Morey C Rougeulle C Avner P 2008 Molecular coupling of Xist regulation and pluripotency. Science 321:1693–1695. doi: 10.1126/science.1160952 18802003

[bib51] Navarro P Oldfield A Legoupi J Festuccia N Dubois A Attia M Schoorlemmer J Rougeulle C Chambers I Avner P 2010 Molecular coupling of Tsix regulation and pluripotency. Nature 468:457–460. doi: 10.1038/nature09496 21085182

[bib52] Navarro P Pichard S Ciaudo C Avner P Rougeulle C 2005 Tsix transcription across the Xist gene alters chromatin conformation without affecting Xist transcription: implications for X-chromosome inactivation. Genes & Development 19:1474–1484. doi: 10.1101/gad.341105 15964997 PMC1151664

[bib53] Nesterova TB Senner CE Schneider J Alcayna-Stevens T Tattermusch A Hemberger M Brockdorff N 2011 Pluripotency factor binding and Tsix expression act synergistically to repress Xist in undifferentiated embryonic stem cells. Epigenetics & Chromatin 4:17. doi: 10.1186/1756-8935-4-17 21982142 PMC3197471

[bib54] Nora EP Lajoie BR Schulz EG Giorgetti L Okamoto I Servant N Piolot T Van Berkum NL Meisig J Sedat J Gribnau J Barillot E Bluthgen N Dekker J Heard E 2012 Spatial partitioning of the regulatory landscape of the X-inactivation centre. Nature 485:381–385. doi: 10.1038/nature11049 22495304 PMC3555144

[bib55] Ohhata T Hoki Y Sasaki H Sado T 2006 Tsix-deficient X chromosome does not undergo inactivation in the embryonic lineage in males: implications for Tsix-independent silencing of Xist. Cytogenetic and Genome Research 113:345–349. doi: 10.1159/000090851 16575199

[bib56] Pauli F 2010 Myers Lab ChIP-seq Protocol, v041610.1 and v041610.2. In: Myers R editor. http://www.hudsonalpha.org/myers-lab

[bib57] Pollex T Heard E 2012 Recent advances in X-chromosome inactivation research. Current Opinion in Cell Biology 24:825–832. doi: 10.1016/j.ceb.2012.10.007 23142477

[bib58] Quinlan AR Hall IM 2010 BEDTools: a flexible suite of utilities for comparing genomic features. Bioinformatics 26:841–842. doi: 10.1093/bioinformatics/btq033 20110278 PMC2832824

[bib59] Raja SJ Charapitsa I Conrad T Vaquerizas JM Gebhardt P Holz H Kadlec J Fraterman S Luscombe NM Akhtar A 2010 The nonspecific lethal complex is a transcriptional regulator in Drosophila. Molecular Cell 38:827–841. doi: 10.1016/j.molcel.2010.05.021 20620954

[bib60] Ramirez F Dundar F Diehl S Gruning BA Manke T 2014 deepTools: a flexible platform for exploring deep-sequencing data. Nucleic Acids Research. Epub ahead of print 10.1093/nar/gku365 PMC4086134 24799436

[bib61] Rougeulle C Avner P 2004 The role of antisense transcription in the regulation of X-inactivation. Current Topics in Developmental Biology 63:61–89. doi: 10.1016/S0070-2153(04)63003-1 15536014

[bib62] Sapountzi V Cote J 2011 MYST-family histone acetyltransferases: beyond chromatin. Cellular and Molecular Life Sciences 68:1147–1156. doi: 10.1007/s00018-010-0599-9 21132344 PMC11114825

[bib63] Shin J Bossenz M Chung Y Ma H Byron M Taniguchi-Ishigaki N Zhu X Jiao B Hall LL Green MR Jones SN Hermans-Borgmeyer I Lawrence JB Bach I 2010 Maternal Rnf12/RLIM is required for imprinted X-chromosome inactivation in mice. Nature 467:977–981. doi: 10.1038/nature09457 20962847 PMC2967734

[bib64] Smith ER Cayrou C Huang R Lane WS Cote J Lucchesi JC 2005 A human protein complex homologous to the Drosophila MSL complex is responsible for the majority of histone H4 acetylation at lysine 16. Molecular and Cellular Biology 25:9175–9188. doi: 10.1128/MCB.25.21.9175-9188.2005 16227571 PMC1265810

[bib65] Splinter E de Wit E Nora EP Klous P Van De Werken HJ Zhu Y Kaaij LJ Van Ijcken W Gribnau J Heard E de Laat W 2011 The inactive X chromosome adopts a unique three-dimensional conformation that is dependent on Xist RNA. Genes & Development 25:1371–1383. doi: 10.1101/gad.633311 21690198 PMC3134081

[bib66] Stadler MB Murr R Burger L Ivanek R Lienert F Scholer A Van Nimwegen E Wirbelauer C Oakeley EJ Gaidatzis D Tiwari VK Schubeler D 2011 DNA-binding factors shape the mouse methylome at distal regulatory regions. Nature 480:490–495. doi: 10.1038/nature10716 22170606

[bib67] Stavropoulos N Lu N Lee JT 2001 A functional role for Tsix transcription in blocking Xist RNA accumulation but not in X-chromosome choice. Proceedings of the National Academy of Sciences of the United States of America 98:10232–10237. doi: 10.1073/pnas.171243598 11481444 PMC56944

[bib68] Sun BK Deaton AM Lee JT 2006 A transient heterochromatic state in Xist preempts X inactivation choice without RNA stabilization. Molecular Cell 21:617–628. doi: 10.1016/j.molcel.2006.01.028 16507360

[bib69] Sun S Del Rosario BC Szanto A Ogawa Y Jeon Y Lee JT 2013 Jpx RNA activates Xist by evicting CTCF. Cell 153:1537–1551. doi: 10.1016/j.cell.2013.05.028 23791181 PMC3777401

[bib70] Taipale M Rea S Richter K Vilar A Lichter P Imhof A Akhtar A 2005 hMOF histone acetyltransferase is required for histone H4 lysine 16 acetylation in mammalian cells. Molecular and Cellular Biology 25:6798–6810. doi: 10.1128/MCB.25.15.6798-6810.2005 16024812 PMC1190338

[bib71] Taylor G Eskeland R Hekimoglu-Balkan B Pradeepa M Bickmore WA 2013 H4K16 acetylation marks active genes and enhancers of embryonic stem cells, but does not alter chromatin compaction. Genome Research 23:2053–2065. doi: 10.1101/gr.155028.113 23990607 PMC3847775

[bib72] Thomas T Dixon MP Kueh AJ Voss AK 2008 Mof (MYST1 or KAT8) is essential for progression of embryonic development past the blastocyst stage and required for normal chromatin architecture. Molecular and Cellular Biology 28:5093–5105. doi: 10.1128/MCB.02202-07 18541669 PMC2519697

[bib73] Thomas-Chollier M Hufton A Heinig M O'keeffe S Masri NE Roider HG Manke T Vingron M 2011 Transcription factor binding predictions using TRAP for the analysis of ChIP-seq data and regulatory SNPs. Nature Protocols 6:1860–1869. doi: 10.1038/nprot.2011.409 22051799

[bib74] Thorvaldsdottir H Robinson JT Mesirov JP 2013 Integrative Genomics Viewer (IGV): high-performance genomics data visualization and exploration. Briefings in Bioinformatics 14:178–192. doi: 10.1093/bib/bbs017 22517427 PMC3603213

[bib75] Tian D Sun S Lee JT 2010 The long noncoding RNA, Jpx, is a molecular switch for X chromosome inactivation. Cell 143:390–403. doi: 10.1016/j.cell.2010.09.049 21029862 PMC2994261

[bib76] Trapnell C Hendrickson DG Sauvageau M Goff L Rinn JL Pachter L 2013 Differential analysis of gene regulation at transcript resolution with RNA-seq. Nature Biotechnology 31:46–53. doi: 10.1038/nbt.2450 PMC3869392 23222703

[bib77] Vigneau S Augui S Navarro P Avner P Clerc P 2006 An essential role for the DXPas34 tandem repeat and Tsix transcription in the counting process of X chromosome inactivation. Proceedings of the National Academy of Sciences of the United States of America 103:7390–7395. doi: 10.1073/pnas.0602381103 16648248 PMC1464350

[bib79] Whyte WA Orlando DA Hnisz D Abraham BJ Lin CY Kagey MH Rahl PB Lee TI Young RA 2013 Master transcription factors and mediator establish super-enhancers at key cell identity genes. Cell 153:307–319. doi: 10.1016/j.cell.2013.03.035 23582322 PMC3653129

[bib80] Wu H Caffo B Jaffee HA Irizarry RA Feinberg AP 2010 Redefining CpG islands using hidden Markov models. Biostatistics 11:499–514. doi: 10.1093/biostatistics/kxq005 20212320 PMC2883304

[bib81] Wu L Zee BM Wang Y Garcia BA Dou Y 2011 The RING finger protein MSL2 in the MOF complex is an E3 ubiquitin ligase for H2B K34 and is involved in crosstalk with H3 K4 and K79 methylation. Molecular Cell 43:132–144. doi: 10.1016/j.molcel.2011.05.015 21726816 PMC4119175

[bib82] Xie X Lu J Kulbokas EJ Golub TR Mootha V Lindblad-Toh K Lander ES Kellis M 2005 Systematic discovery of regulatory motifs in human promoters and 3' UTRs by comparison of several mammals. Nature 434:338–345. doi: 10.1038/nature03441 15735639 PMC2923337

[bib83] Yoshida K Toki T Okuno Y Kanezaki R Shiraishi Y Sato-Otsubo A Sanada M Park MJ Terui K Suzuki H Kon A Nagata Y Sato Y Wang R Shiba N Chiba K Tanaka H Hama A Muramatsu H Hasegawa D Nakamura K Kanegane H Tsukamoto K Adachi S Kawakami K Kato K Nishimura R Izraeli S Hayashi Y Miyano S Kojima S Ito E Ogawa S 2013 The landscape of somatic mutations in Down syndrome-related myeloid disorders. Nature Genetics 45:1293–1299. doi: 10.1038/ng.2759 24056718

[bib84] Young RA 2011 Control of the embryonic stem cell state. Cell 144:940–954. doi: 10.1016/j.cell.2011.01.032 21414485 PMC3099475

[bib85] Zawel L Dai JL Buckhaults P Zhou S Kinzler KW Vogelstein B Kern SE 1998 Human Smad3 and Smad4 are sequence-specific transcription activators. Molecular Cell 1:611–617. doi: 10.1016/S1097-2765(00)80061-1 9660945

[bib86] Zhang Y Liu T Meyer CA Eeckhoute J Johnson DS Bernstein BE Nusbaum C Myers RM Brown M Li W Liu XS 2008 Model-based analysis of ChIP-Seq (MACS). Genome Biology 9:R137. doi: 10.1186/gb-2008-9-9-r137 18798982 PMC2592715

[bib87] Zhao W Li Q Ayers S Gu Y Shi Z Zhu Q Chen Y Wang HY Wang RF 2013a Jmjd3 inhibits reprogramming by upregulating expression of INK4a/Arf and targeting PHF20 for ubiquitination. Cell 152:1037–1050. doi: 10.1016/j.cell.2013.02.006 23452852 PMC3742052

[bib88] Zhao X Su J Wang F Liu D Ding J Yang Y Conaway JW Conaway RC Cao L Wu D Wu M Cai Y Jin J 2013b Crosstalk between NSL histone acetyltransferase and MLL/SET complexes: NSL complex functions in promoting histone H3K4 di-methylation activity by MLL/SET complexes. PLOS Genetics 9:e1003940. doi: 10.1371/journal.pgen.1003940 24244196 PMC3828133

[bib89] Zollino M Orteschi D Murdolo M Lattante S Battaglia D Stefanini C Mercuri E Chiurazzi P Neri G Marangi G 2012 Mutations in KANSL1 cause the 17q21.31 microdeletion syndrome phenotype. Nature Genetics 44:636–638. doi: 10.1038/ng.2257 22544367

